# Lipopeptide Engineering: From Natural Origins to Rational Design Against Antimicrobial Resistance

**DOI:** 10.3390/antibiotics15010100

**Published:** 2026-01-19

**Authors:** Shi-Yu Xie, Fang-Jing He, Ying-Ying Yang, Yan-Fei Tao, Xu Wang

**Affiliations:** 1 National Reference Laboratory of Veterinary Drug Residues (HZAU), Huazhong Agricultural University, Wuhan 430070, China; xieshiyu@webmail.hzau.edu.cn (S.-Y.X.); hefangjing@webmail.hzau.edu.cn (F.-J.H.); yangyingying@webmail.hzau.edu.cn (Y.-Y.Y.); 2 MAO Key Laboratory for Detection of Veterinary Drug Residues, Huazhong Agricultural University, Wuhan 430070, China

**Keywords:** lipopeptides, resistance, rational design and optimization, non-ribosomal peptide synthetase (NRPS) engineering, targeted delivery systems, synthetic bioinformatic natural product, artificial intelligence (AI)-driven discovery

## Abstract

Lipopeptides (LPs) have evolved from naturally occurring compounds to key therapeutic agents against multidrug-resistant (MDR) bacterial infections. However, their expanding clinical use has triggered emerging resistance mechanisms, posing serious challenges to anti-infective therapy. This systematic review outlines the development of LP resistance and highlights innovative strategies to counteract it. To overcome these evolving barriers, the field has transitioned from traditional empirical optimization to multidimensional rational design. Moving beyond conventional structure–activity relationship (SAR)-guided chemical synthesis, current approaches integrate diverse innovative methodologies. Based on these advances, this review provides the first systematic summary of contemporary strategies for developing novel LPs, offering new perspectives and methodological support to combat resistant bacterial infections and accelerate the development of next-generation LP-based therapeutics.

## 1. Introduction

### 1.1. Structural Composition and Biological Function of LPs

Lipopeptides (LPs) are a class of amphiphilic bioactive molecules produced by microorganisms via nonribosomal peptide synthetase (NRPS) or post-ribosomal modification pathways. Their typical structure consists of a hydrophobic fatty acid (FA) linked to a hydrophilic peptide moiety [1]. Their biosynthesis primarily relies on the NRPS machinery, and their bioactivity is modulated by factors including lipid chain length, peptide ring size, and amino acid modifications. Differences among LP families are mainly reflected in structural variations and target specificity, where diversity in the peptide sequence and FA contributes to distinct host targeting and biological activities. Thus, structural differences underpin their specific functions and application potential, providing a diverse molecular foundation for drug development and agricultural biocontrol strategies.

### 1.2. Advantages of LPs Targeting MDR Bacteria

The accelerating spread of antimicrobial resistance (AMR) worldwide further fuels the demand for novel antimicrobial agents [2,3]. LPs offer significant advantages in treating drug-resistant bacterial infections. Their mechanism of action, disrupting bacterial cell membranes (CMs), leading to rapid bactericidal effects, makes it difficult for bacteria to develop resistance [4]. Furthermore, LPs can penetrate biofilms, overcoming the limitations of conventional antibiotics in chronic infections [5,6]. Although LPs are indispensable first-line drugs for combating resistant bacteria, their clinical use remains constrained by issues of safety, stability, targeting specificity, and inherent resistance. For instance, polymyxins are associated with nephrotoxicity [7,8] and neurotoxicity [9], and daptomycin (DAP) carries a risk of myotoxicity [10]. The nephrotoxicity of LPs has been linked to their ability to induce apoptosis in renal tubular cells and cause DNA damage, leading to genomic instability [11,12]. Additionally, neurotoxicity can be mediated through a significant increase in acetylcholinesterase activity [13]. Surfactin interacts with erythrocyte membrane phospholipids via a “carpet” or “pore-forming” model, increasing membrane permeability, inhibiting ATPase activity, and ultimately causing hemolysis. Additionally, antimicrobial peptides (AMPs) tend to bind phosphatidylserine on host cells, resulting in mammalian cell cytotoxicity. LPs also struggle to penetrate the outer membrane (OM) barrier of Gram-negative (G^−^) bacteria [14]. Furthermore, the spread of resistance mediated by bacterial genetic mutations and adaptive evolution has become increasingly severe, with resistant strains to LP already emerging. Examples include *mcr-1*-mediated polymyxin resistance, where the *mcr-1*-encoded phosphor ethanolamine transferase modifies lipopolysaccharide (LPS), reducing polymyxin B (PMB) binding [15], and *Staphylococcus aureus* resistance through *mprF* mutations that increase membrane phosphatidyl lysine synthesis, altering membrane charge and decreasing adsorption of cationic LPs [16].

Given the challenges associated with LPs in clinical settings—including toxicity, pharmacokinetic limitations, and the risk of drug resistance—the quest for safer, more targeted, and less resistance-prone LPs is of paramount importance. It is well recognized that de novo drug discovery is an exceptionally arduous process. Therefore, a more efficient strategy involves the structural optimization of existing LPs by leveraging advanced technologies. This approach aims to overcome the aforementioned drawbacks while facilitating the rapid clinical translation of improved candidates. Accordingly, this review focuses on discussing the optimization and engineering strategies for LPs.

### 1.3. Innovative Strategies for LP Optimization

To overcome these challenges, researchers are actively exploring innovative strategies for designing next-generation LP-based therapeutics. These approaches include: structural modification of existing LPs via semi-synthesis or medicinal chemistry to improve their safety and efficacy; engineering of LP biosynthetic gene clusters (BGCs) by reprogramming substrate specificity, recombining modules, or modifying enzymatic functions to enable efficient biosynthesis of novel analogs; and the development of advanced targeted delivery systems, particularly nanoparticle-based carriers, to enhance tissue-specific accumulation [17,18] and reduce off-target effects [19,20,21]. The deep integration of NRPS engineering with AI and high-throughput screening (HTS) technologies—such as using Transformer-based protein language models to predict and generate NRPS activities, combined with picoliter-scale ultra-HTS—has enabled rapid identification of high-performance LPs from libraries comprising tens of millions of molecules [22]. Against this backdrop, this review summarizes recent advances in rational structural redesign, NRPS combinatorial biosynthesis, intelligent drug delivery platforms, and AI-driven LP design. By integrating insights from mechanistic studies, resistance genetics, and pharmaceutical technologies, we aim to provide a comprehensive perspective for developing more effective and safer LP-based antibiotics to combat the escalating AMR crisis.

## 2. Renewal and Upgrading of LPs

The discovery and iterative refinement of LPs have advanced in parallel with innovations in their development technologies. The history of LP development is, in essence, a chronicle of evolving strategies to counteract bacterial resistance. Since the discovery of the first LP, the field has evolved through several distinct stages: the initial isolation of natural LPs, followed by SAR-guided modification, the advent of NRPS engineering, and the emergence of machine learning (ML)-enabled rational design.

### 2.1. Discovery of Natural LPs

**Figure 1:**
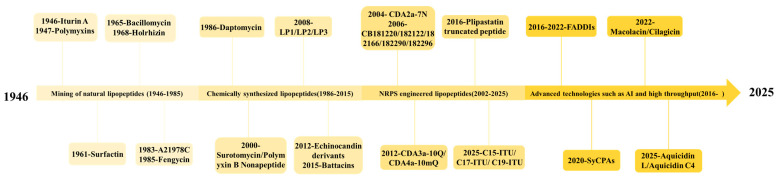
Evolution of Novel LPs. A brief timeline delineating the development of technologies for LP discovery across different eras, aligned with the introduction of new LP classes. The horizontal axis divides the progression into distinct technological eras, while corresponding milestones for the introduction of novel LPs are indicated above and below the timeline.

The early discovery of natural LPs received insufficient attention. However, with the clinical emergence of superbugs and the critical shortage of effective antibiotics, researchers have redirected their focus to the discovery and application of evolutionarily guided bacterial LPs. This shift offers a breakthrough therapeutic strategy for combating current drug-resistant infections (Figure 1). Most natural LPs are synthesized and secreted by *Bacillus*, *Paenibacillus* spp., and *Streptomyces* (Table 1) [23,24]. Most LPs exhibit remarkable structural and functional diversity, along with potent biological activities, making them valuable resources for the development of clinical antibiotics or lead compounds [24]. Notably, polymyxins and DAP provide privileged natural structural scaffolds for combating G^−^ and Gram-positive (G^+^) bacteria, respectively. Furthermore, LPs such as surfactin, iturin, and fengycin exhibit versatile bioactivities with applications spanning medicine, food preservation, industry, and agriculture, underscoring their outstanding potential for development.

The LP-surfactin was first isolated by Soviet scientists in the 1940s. However, its therapeutic potential was largely unrecognized at the time [25]. Subsequently, PMB and colistin were found to exert their specific antibacterial effects by targeting LPS, disrupting the outermost permeability barrier—the OM—of G^−^ bacteria, and subsequently damaging the CM [26]. Furthermore, PB can inhibit the nicotinamide adenine dinucleotide hydride oxidases in the electron transport chain and induce the production of reactive oxygen species (ROS), ultimately resulting in cell death [27]. It is precisely because of its unique antibacterial mechanism that colistin exerts potent lethal effects against G^−^ bacteria, including MDR *Klebsiella pneumoniae*, *Acinetobacter baumannii*, *Escherichia coli*, and *Pseudomonas aeruginosa* (Table 1) [28,29]. Consequently, it is regarded as the “last line of defense” against such infections. However, due to significant dose-limiting nephrotoxicity and neurotoxicity, their clinical application declined after the 1970s [30]. In recent years, research efforts have been directed toward developing polymyxin derivatives, such as FADDI-002, SPR206, MRX-8, Disulfide-linked analog 18b, many of which demonstrate improved therapeutic efficacy and pharmacokinetic properties compared to the parent compounds (Table 1) [31].

**Table 1:** The antibacterial activity (MIC: μg/mL) and cytotoxicity (μg/mL) of polymyxin derivatives.

Polymyxin Derivatives	Structure	MIC (μg/mL)				Cytotoxicity (μg/mL)	References
		*K. pneumoniae*	*A. baumannii*	*P. aeruginosa*	*E. coli*		
Polymyxin A/B/D/E/M/S	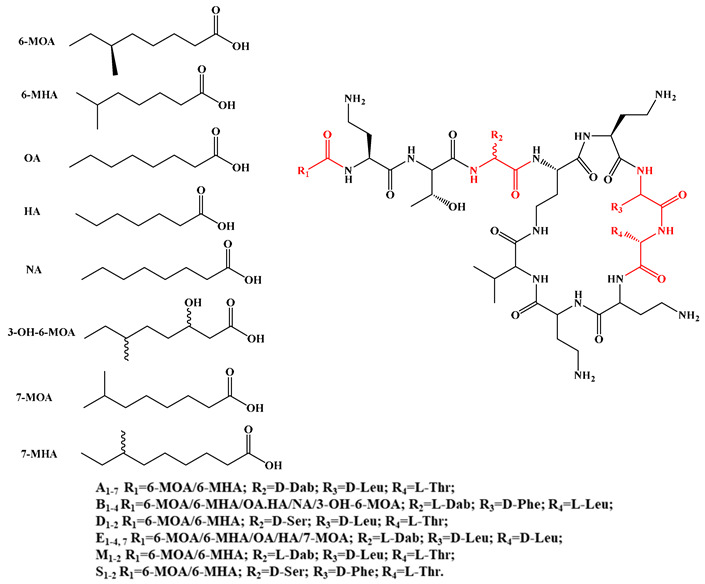	0.25–2	0.06–2	0.5–4	0.5–2	33–500	[32]
Colistin	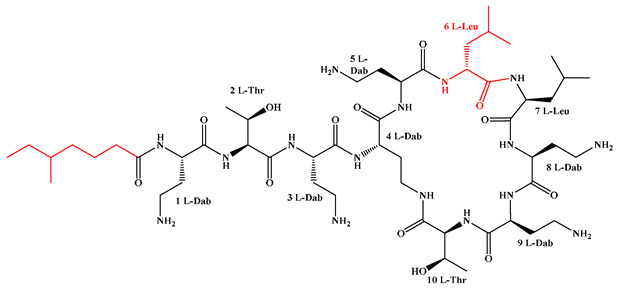	N.D.	N.D.	0.25–2	N.D.	N.D.	[33,34]
SPR741	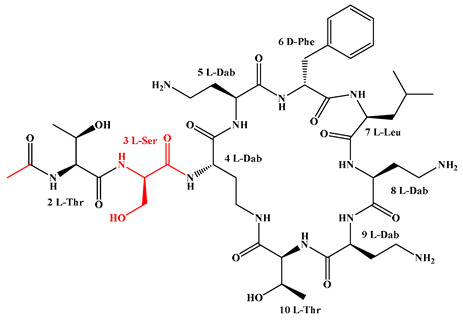	>64	64	32	32 *	N.D.	[35]
KA1	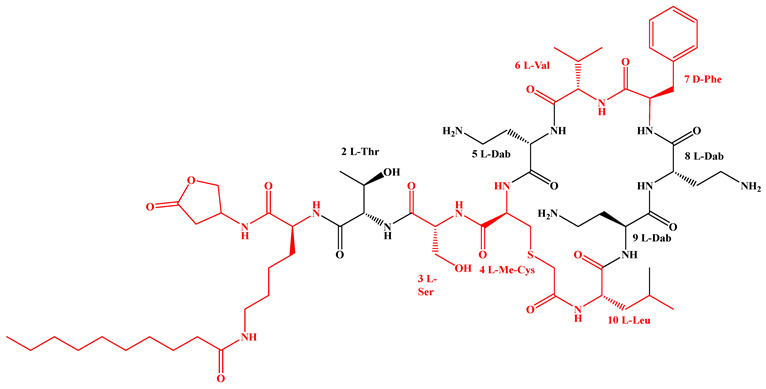	1	1	N.D.	1	>16	[36]
KA2	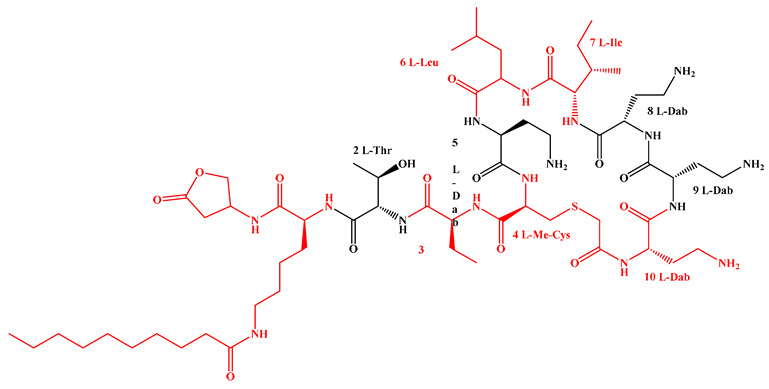						
FADDI-287	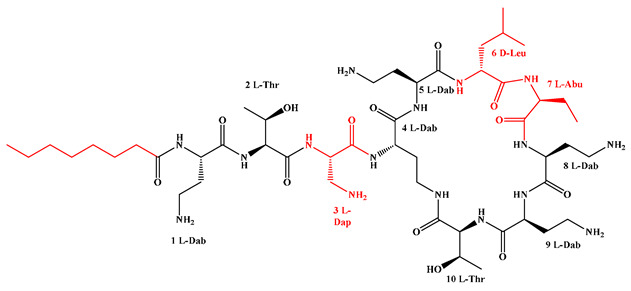	64 (64) *	16 (8) *	64 (32) *	8 (64) *	N.D.	[37]
FADDI-002	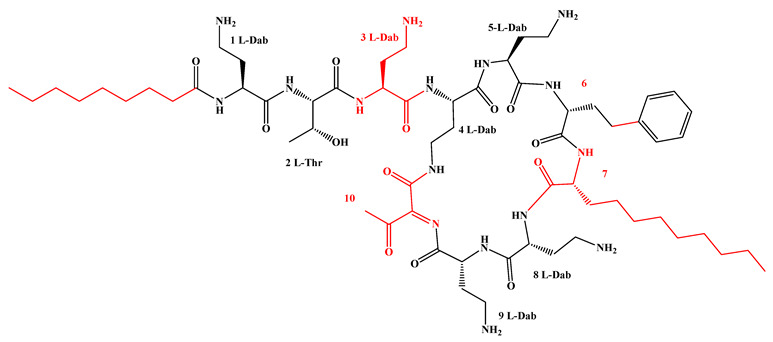	0.25 (0.25)	0.5 (16) *	0.25–1 (32–64) *	1–2 (0.125–2) *	≈1280	[33]
FADDI-003	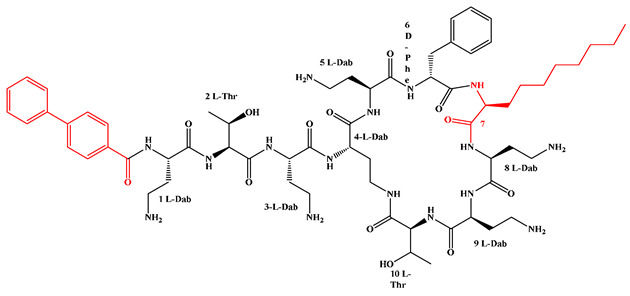	N.D.	2–16	N.D.	N.D.	N.D.	[38]
CA824	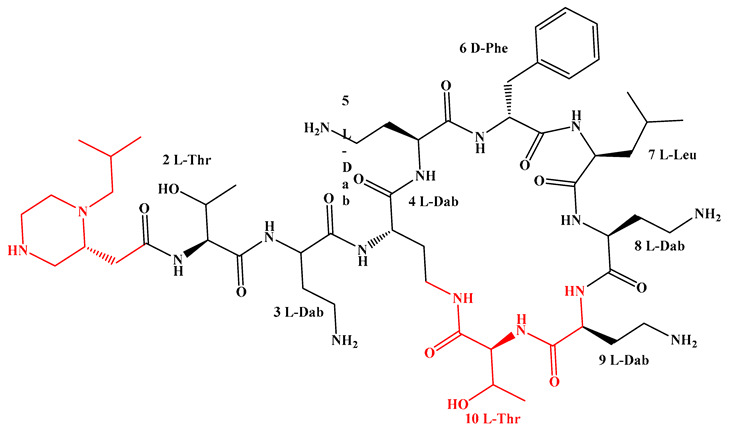	N.D.	N.D.	N.D.	N.D.	148	[31]
NAB739	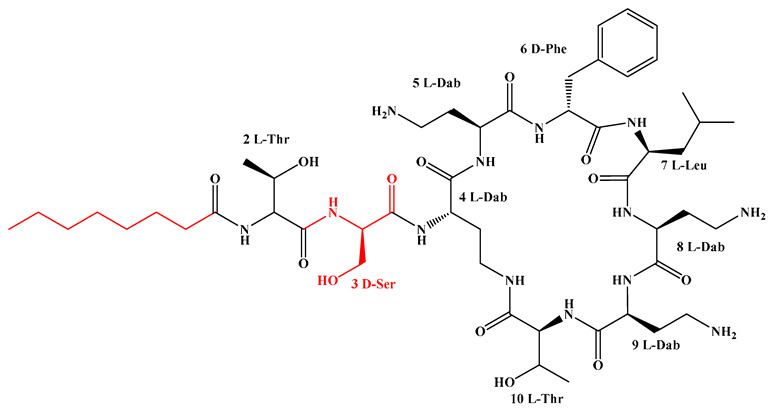	2–4 (0.5–1)	1–8 (0.5–1)	16 (2)	0.5–2 (0.25–1)	237	[39,40]
NAB815	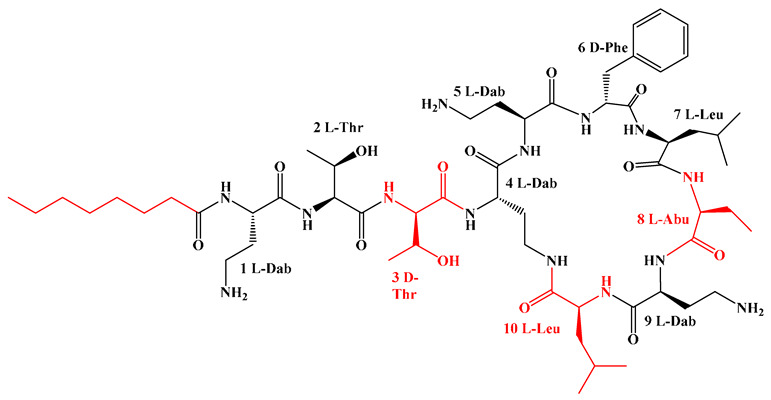	2–8 (0.5–1)	0.5–4 (0.5–1)	64 (2)	0.5–4 (0.25–1)	334	[40]
SPR206 (Upleganan)	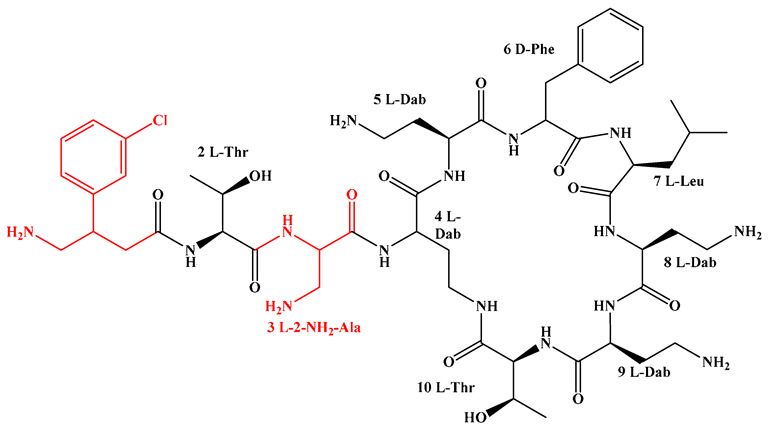	N.D.	0.03–4 * (0.5–256)	N.D.	N.D.	N.D.	[34]
MRX-8	N.D.	0.12	0.5	0.5	0.12	N.D.	[41,42]
Disulfide-linked analog 18b	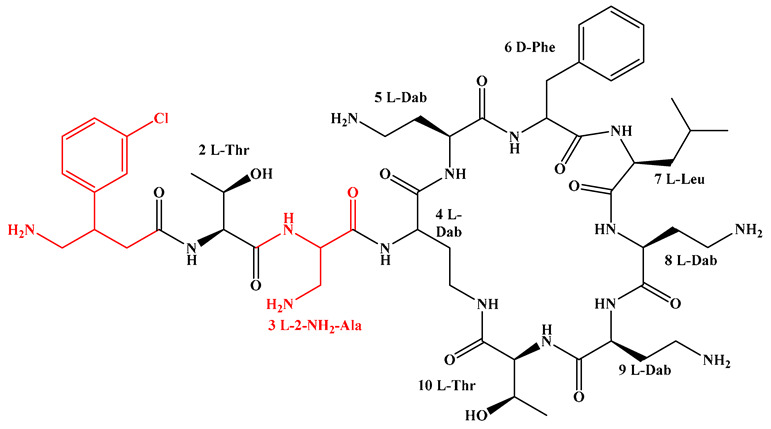	0.25 (0.25)	0.25–1 (0.25)	1 (0.5–1)	1 (1)	192 μM (41 μM)	[43]
Non-disulfide containing analog 45	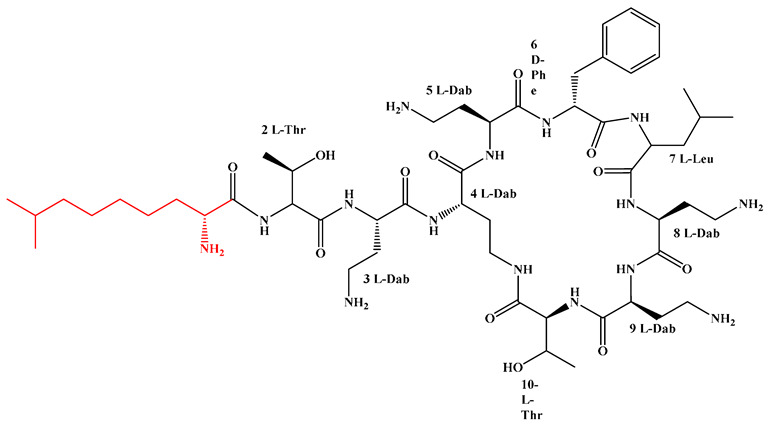	≤0.25 (0.25)	0.25–0.5 (0.25)	1 (0.5–1)	1 (1)	82 μM (41 μM)	[43]
EB12	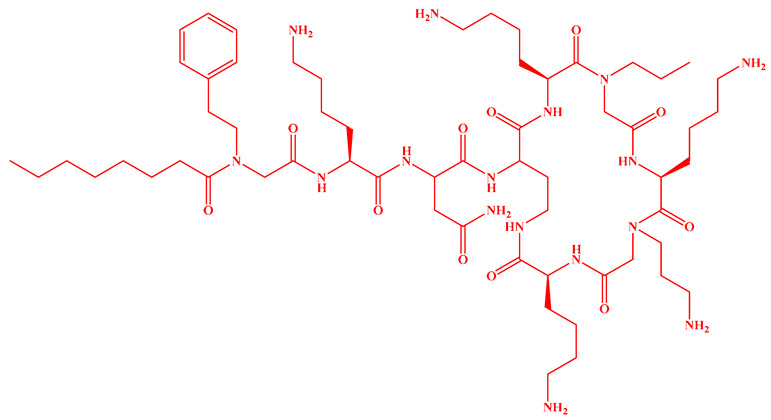	N.D.	N.D.	2 (0.25–1)	32 (1)	>150	[44]
EB13	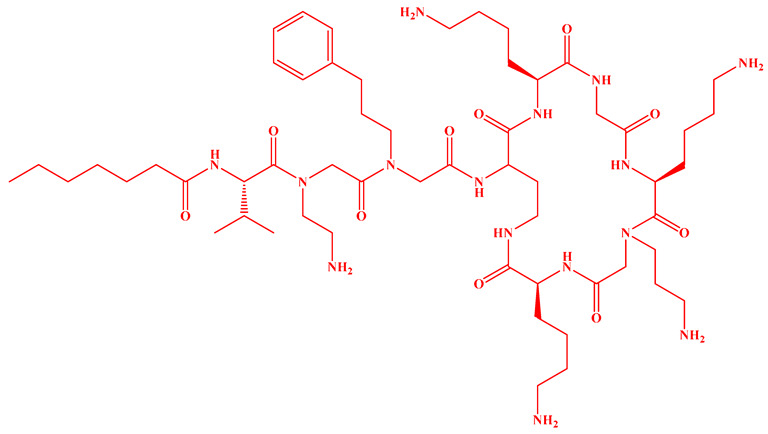						
CEP936	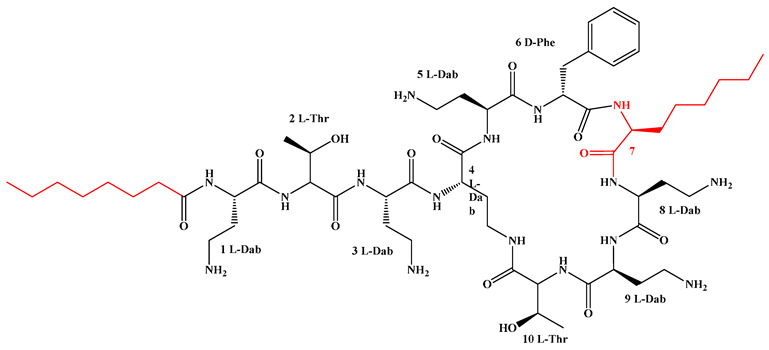	N.D.	N.D.	0.5–32/0.25–8 (0.125–64) *	N.D.	N.D.	[45]
CEP938	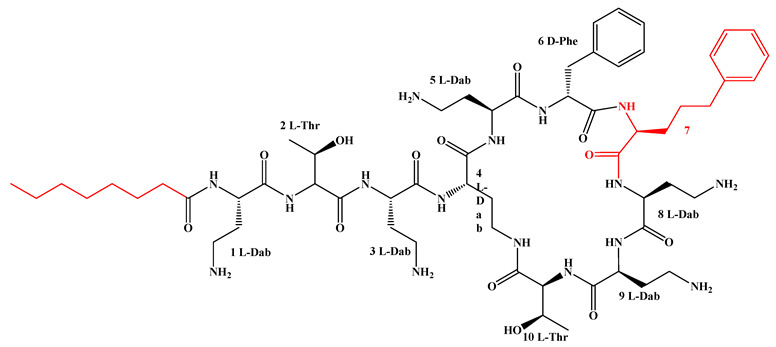						
Semisynthetic LP derivative-5 v	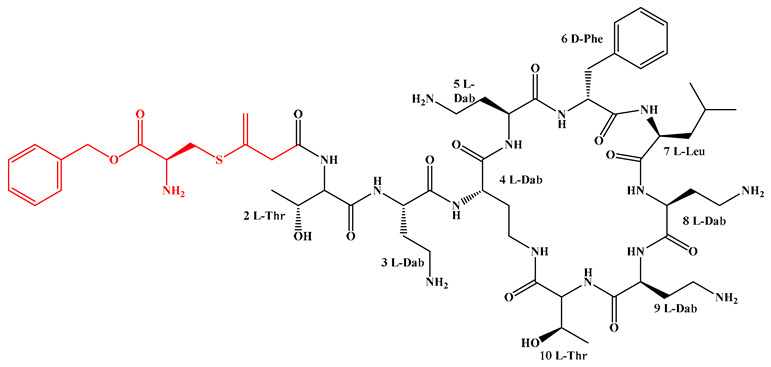	1 (1)	0.125 (0.25)	0.25 (0.5)	0.25 (0.125)	>300	[46]
Polymyxin-*N*-terminal	N.D.	8 -> 64 (0.25)	0.5–4 (16) *	16 -> 64 (32–64) *	4–32 (0.125–2) *	<450 (>1280)	[33]
Polymyxin-P6/P7	N.D.	<0.125–16 (0.25)	<0.25–32 (16) *	0.125–2 (32–64) *	<0.125–16 (0.125–2) *	<450 (>1280)	[33]
Bip-macolacin	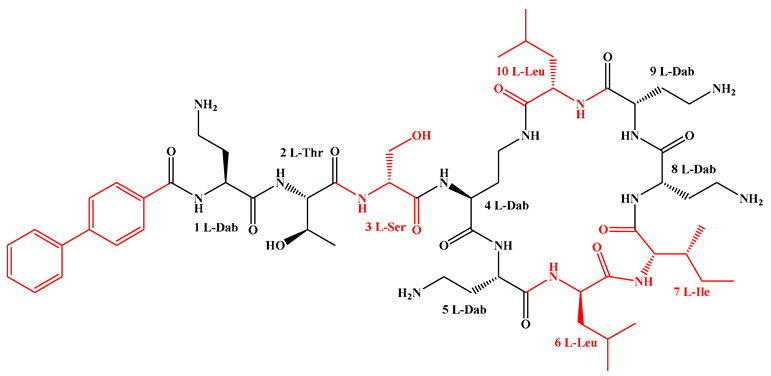	2–4 (0.25)	0.25–0.5 (16) *	1–2 (32–64) *	4–8 (0.125–2) *	>1280	[33]

* Represents clinical isolation of MDR; () contains polymyxin B or colistin MIC; N.D. indicates no data.

Another major milestone was achieved in the 1980s when Eli Lilly isolated the A21978C complex from *Streptomyces roseosporus*, which later served as the precursor for the development of DAP through structural optimization [47,48]. DAP exerts its effect against G^+^ bacteria, including multidrug-resistant *S. aureus* (MRSA), *Enterococcus faecium*, and *Streptococcus* species, through a mechanism distinct from that of polymyxins. Its antibacterial activity is strictly calcium-dependent: at physiological concentrations of Ca^2+^, DAP undergoes a structural transition. The activated molecule inserts its lipid tail into regions of the bacterial CM enriched with phosphatidylglycerol (PG) and cardiolipin (CL), while its cyclic peptide structure electrostatically associates with the negatively charged membrane surface [49,50,51]. Approved by the U.S. Food and Drug Administration in 2003 under the brand name Cubicin^®^, DAP became the first cyclic LP for treating drug-resistant G^+^ pathogens. Its unique calcium-dependent membrane-targeting mechanism circumvents cross-resistance with conventional antibiotic targets [52]. However, clinical use revealed limitations such as myotoxicity, interference with pulmonary surfactant, and the emergence of resistance in certain strains, prompting the need for further structural optimization [10,51]. Subsequent research has focused on modifying the lipid tail, amino acid sequence, and cyclic architecture of the natural LP, leading to the development of safer and more potent derivatives. A notable example is surotomycin, a DAP analog designed for treating *Clostridium difficile* infections [53] (Table 2).

### 2.2. Limitations of Existing LPs

The amphiphilic structure of LPs enables them to insert into and disrupt phospholipid-rich biological membranes in a detergent-like manner [61]. Their antibacterial selectivity stems from a higher affinity for bacterial membranes rich in anionic phosphatidylglycerol compared to the electrically neutral phosphatidylcholine membranes of mammalian cells [62,63]. However, this selectivity is relative; at high concentrations or upon prolonged exposure, nonspecific effects on host cell membranes can still occur [64]. Clinically, the effective bactericidal concentration of some LPs is often close to the concentration that induces host toxicity, resulting in a narrow therapeutic window. For instance, as mentioned in Section 2.1, the nephrotoxicity and neurotoxicity of polymyxins and the myotoxicity of daptomycin necessitate strict dosing limits and upper thresholds for plasma concentrations. The cationic structure of polymyxins allows them to directly adhere to and disrupt the integrity of renal tubular cell membranes, also inducing oxidative stress and apoptosis [65]. The effective steady-state plasma concentration for polymyxin E is approximately 2 mg/L, while the risk factor for nephrotoxicity is a trough concentration > 2.3 mg/L, indicating a near overlap [66,67,68]. The therapeutic window for daptomycin varies significantly among individuals and must be dynamically defined using PK/PD targets, managed through therapeutic drug monitoring and clinical observation as a personalized concentration range [69]. In fact, most naturally occurring or engineered LPs exhibit cytotoxicity and hemolytic toxicity when administered in their native form in vivo, as reported for natural LPs like Surfactins, Lichenysins, and Echinocandin B, as well as engineered variants such as Plipastatin and C15-ITU/C17-ITU/C19-ITU (Table 3). Due to their amino acid structure, LPs may interact with host proteins/peptides and possess potential immunogenicity, which could trigger allergic reactions [70,71]. Furthermore, during in vivo application, they face degradation by proteolytic enzymes in the digestive tract (e.g., trypsin, chymotrypsin) and tissue peptidases, leading to reduced efficacy compared to in vitro results and resulting in poor bioavailability [72,73]. Therefore, addressing the current clinical limitations of LPs requires structural optimization of existing lead compounds.

### 2.3. Novel LPs Based on New Methods

Building upon existing knowledge and propelled by technological advances, more sophisticated approaches are now being employed to develop LPs. For instance, NRPS engineering enables the rational design of LP derivatives to combat drug-resistant bacteria or enhance therapeutic efficacy. This has been demonstrated by the use of CRISPR-Cas9 gene editing to rapidly redesign the synthetase gene cluster for the LP enduracidin [74], as well as by targeted knockout and plasmid-based complementation of NRPS/polyketide synthase systems to improve the synthesis of Iturin A [75]. Furthermore, given that many unculturable bacteria represent a rich natural reservoir of LPs, bioinformatics-guided mining combined with chemical synthesis has been applied to explore these strains. This strategy has led to the discovery of LPs such as macolacin and cilagicin, which demonstrate promising efficacy against drug-resistant pathogens [76,77,78]. ML has significantly accelerated the development of AMPs. Analyzing large-scale databases enables the low-cost, high-efficiency screening and design of AMPs [79]. Furthermore, it can guide the evolutionary prediction and enhancement of natural peptides for greater efficacy. For example, Wang et al. utilized explainable AI to direct the evolution of natural AMP sequences, successfully improving their antibacterial potency [80]. Separately, the Coelho team employed ML to predict novel AMPs from large-scale metagenomic and genomic datasets, constructing the AMP Sphere resource. Experimental validation confirmed that a significant portion of the corresponding synthetic peptides exhibited membrane-disruptive antimicrobial activity against drug-resistant pathogens [81].

This paradigm shift, driven by AMR and moving from mere discovery to rational design, represents a veritable molecular “arms race” against bacteria. To gain a decisive advantage in this contest, it is imperative to precisely decipher the complex and diverse resistance mechanisms that bacteria have evolved against LPs.

**Table 3:** The Development History of LPs.

Year	Representative LPs	Source	Major Function	Mechanism of Action	Limitations	References
1946	Iturins	*Bacillus subtilis*	Anti-G^+^ Anti-fungal	Binds to ergosterol in the fungal CM via β-amino acid chains, forming ion channels that disrupt membrane integrity	Hemolytic	[82,83]
1947	Colistins/Polymyxins	*Paenibacillus polymyxa*	Anti-G^−^	Positively charged diaminobutyric acid (Dab) residues electrostatically interact with the negatively charged phosphate groups of lipid A	Nephrotoxicity, Neurotoxicity	[26,84,85,86]
1961	Surfactins	*B. subtilis*	Surfactant Antiviral Anti-G^−^	Forming non-specific ion channels or pores in the membrane	Hemolytic toxicity, Cell damage	[25,87,88]
1965	Bacillomycins	*B. subtilis*	Anti-fungal	Disrupting fungal CMs through ergosterol binding	Low toxicity to bacteria and mammalian cells	[89,90,91]
1968	Laspartomycin	*Streptomyces viridochromogenes*	Anti-G^+^	Membrane destruction and depolarization	High doses cause muscle toxicity	[92]
1985	A21978C	*S. roseosporus*	Anti-G^+^	Membrane damage	Muscle toxicity; Kidney toxicity	[47]
1985	Fengycin	*Bacillus amyloliquefaciens* FZB42	Anti-fungal, Anti-G^+^	Targets fungal CMs containing ergosterol-forming pores and disrupting membrane integrity	Low hemolytic toxicity	[93,94,95]
1986	DAP	Semi-synthetic modification of A21978C	Anti-G^+^	Exerts bactericidal effects through calcium-dependent membrane depolarization and by impeding the binding of proteins *MurG*, *PlsX* to fluid lipids, causing their mislocalization and functional impairment.	Muscle toxicity	[4,96,97,98]
1988	Glidobactins	*Burkholderia* spp.	Anti-G^+^ Anti-fungal, Anticancer	Irreversibly inhibits the proteasome and interferes with the degradation process of proteins within cells.	n. s.	[99,100]
1990/2019	A54145/A54145B	*Streptomyces fradiae*	Anti-G^+^	In the presence of Ca^2+^, it forms membrane pores by binding to specific components, resulting in depolarization and cell death.	n. s.	[101,102]
1995	Lichenysins	*Bacillus licheniformis*	Anti-G^+^	Exhibits excellent surface activity.	High concentration leads to hemolysis	[103,104]
1995	Echinocandin B	*Aspergillus rugulosus*	Anti-fungal	Noncompetitively inhibits 1,3-β-D-glucan synthase by binding to a specific allosteric site on the membrane-bound enzyme complex.	Hemolysis	[105]
2000	Surotomycin	DAP derivatives	Anti-G^+^	Mediating calcium-dependent membrane disruption in vegetative cells.	No significant safety concerns, but low oral bioavailability	[54,106]
2000	PMB Nonapeptide	Polymyxin derivatives	Anti-G^−^	Binds to LPS to increase OM permeability, enhancing the efficacy of hydrophobic antibiotics against G^−^ bacteria.	n. s.	[107]
2004	CDA2a-7N	NRPS engineering	n. s.	n. s.	n. s.	[108]
2005	Putisolvin I/II	Pseudomonas putida PCL1445	Surfactant Anti-G^−^	inhibit biofilm formation and to break down existing biofilms of several *Pseudomonas* spp.	n. s.	[109]
2006	CB181220/182122/182166/182290/182296	NRPS engineering	Anti-G^+^	n. s.	n. s.	[57]
2010	SPR741 (NAB 741)	Chemical synthesis	Antibiotic enhancers, Anti-G^−^	Penetrates G^−^ bacteria OM.	No nephrotoxicity	[110,111]
2012	Pelgipeptin	*Paenibacillus elgii* B69	Anti-G^−^ Anti-G^+^ Anti-fungal	Loss of membrane integrity via phospholipid destruction results in leakage of intracellular components.	Low toxicity	[112]
2012	CDA3a-10Q/CDA4a-10mQ	NRPS engineering	n. s.	n. s.	n. s.	[113]
2012	Echinocandin derivants	Chemical synthesis	Anti-fungal	Hydrophilic amino acids were favored at the “left” tripeptide segment (A–C) of the cyclo-lipo-hexapeptide scaffold, whereas the “right” lipo-tripeptide segment (D–G) was preferred as a hydrophobic core.	n. s.	[114]
2015	Battacins	Chemical synthesis	Anti-G^+^ Anti-G^−^	Inhibit the formation of biofilms by penetrating the extracellular polymer matrix.	Hemolytic toxicity is negligible	[115]
2016	FADDI-019	Chemical synthesis	Anti-G^+^ Anti-G^−^	Increasing the hydrophobic range of the molecule at the 6th position broadens the antibacterial spectrum.	n. s.	[116]
2017	NAB739/NAB815	Chemical synthesis	Anti-G^−^	The same as PMB.	Lower nephrotoxicity compared to PMB	[117]
2018	FADDI-003	Chemical synthesis	Anti-G^−^	Possesses a greater hydrophobic character compared to polymyxins.	n. s.	[38]
2018	Plipastatin (fengycin) derivatives	NRPS engineering	Anti-fungal Anti-G^+^	n. s.	n. s.	[118]
2020	SyCPAs	Synthetic-Bioinformatic Natural Product (Syn-BNP)	Anti-G^+^ Anti-G^−^	Cell lysis by the broad-spectrum SyCPAs 12, 102, and 123; Inhibition of cell wall (CW) biosynthesis by SyCPA 4; Membrane depolarization and Mycobacterium tuberculosis growth inhibition by SyCPA 63.	n. s.	[119]
2021	Glidobactin derivant	*Burkholderia* spp FA (modification)	Anti-G^+^ Anti-fungal Anticancer	n. s.	n. s.	[120,121]
2021	FADDI-287	Chemical synthesis	Anti-G^−^	Induces greater membrane destabilization than polymyxin.	n. s.	[122]
2022	F365 (QPX9003)	Chemical synthesis	Anti-G^−^	Induces greater membrane destabilization than polymyxin.	No observed acute or renal toxicity at therapeutic levels	[123]
2022	Autucedines A–C	*Streptomyces olivaceus* SCSIO T05	n. s.	n. s.	n. s.	[124]
2022	Macolacin	Syn-BNP	Anti-G^−^	The cationic segment disrupts OM integrity via binding to anionic LPS in G^−^ bacteria.	Structural homology to colistin suggests potential nephrotoxicity and neurotoxicity.	[76]
2022	Cilagicin	Syn-BNP	Anti-G^+^	Inhibits CW synthesis by dual targeting of essential lipid carriers (undecaprenyl phosphate and undecaprenyl pyrophosphate).	Nontoxicity	[77]
2023	FADDI-235/236	Chemical synthesis	Anti-G^−^	Minimizing the interaction with phospholipids facilitates polymyxin penetration through the monolayer, resulting in enhanced efficacy.	n. s.	[125]
2023	Chimeric plipastatin/surfactin NRPS for the production of novel peptides	NRPS engineering	Anti-fungal, Anti-G^+^	n. s.	n. s.	[126]
2023	Plipastatin	NRPS engineering	Anti-fungal, Anti-G^+^	It damages the integrity of the fungal CM and inhibits the growth of mycelium. Phospholipase A2, phospholipase C, and phospholipase D of bacteria are inhibited, interfering with cellular signal transduction and metabolism.	Immunosuppressive and cytotoxic at high concentrations	[127]
2025	C15-ITU/ C17-ITU/ C19-ITU	NRPS engineering	Anti-fungal	n. s.	Hemolysis	[128]
2025	Aquicidins	Syn-BNP	Aquicidine C4: Anti-G^+^ Aquicidine L: Anti-G^−^	The linear peptide aquicidine L showed potent anti-G^−^ bacteria activity by mainly targeting both anionic LPS and phosphatidylethanolamine (PE) in the bacterial membrane. The cyclopeptide aquicidine C4 showed potent anti-G^+^ activity by mainly binding to both anionic CL and PG in the membrane.	n. s.	[78]

n. s.: not shown.

## 3. Mechanisms and Solutions of LP Resistance

**Figure 2:**
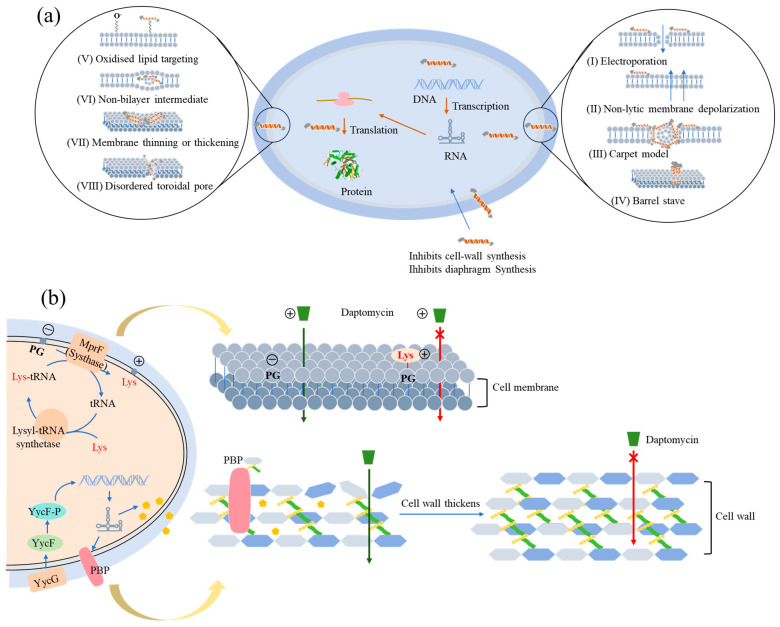
Antibacterial and resistance mechanisms of LPs. (**a**) Primary modes of antibacterial action. Key mechanisms include: inhibition of CW synthesis; membrane disruption—via peptide-induced pore formation, non-lytic membrane depolarization, carpet model, barrel stave, oxidized lipid targeting, non-bilayer intermediate, membrane thinning or thickening, and disordered toroidal pore; and interference with intracellular targets (DNA/RNA/protein synthesis) [131]. (**b**) Example of bacterial resistance to DAP. The diagram depicts resistance conferred by membrane protein *MprF* and the CW-associated *YycF*/*G* two-component system [132].

Owing to their potent efficacy and unique mechanisms of action, LPs are deployed against MDR pathogens. The structural diversity of LPs underpins their functional versatility. For example, they can directly disrupt microbial membrane integrity by forming ion channels [129], specifically recognize CW synthesis precursors like lipid II [77], or bind to specific membrane components such as ergosterol [83] and LPS [130]. This multi-target action reduces the likelihood of resistance development (Figure 2a). Despite this significant potential in combating resistant bacteria, intense selective pressure has driven the evolution of diverse bacterial adaptive mechanisms against LPs. These include bacterial membrane modification, activation of drug efflux pumps, CW thickening, and biofilm formation (Table 4).

### 3.1. Membrane Modification

#### 3.1.1. Membrane Charge Remodeling Approaches to Overcome Resistance

Resistance to LPs arises from coordinated, multi-gene mechanisms. Key systems such as *mprF*, *cls*, *mcr-1*, and the PmrAB/PhoPQ two-component pathways alter phospholipid composition and reduce the net negative surface charge, thereby weakening electrostatic adsorption and binding of cationic LPs.

Resistance to DAP is primarily mediated by *MprF*, *Cls*, *RpoB*, and *RpoC*. DAP binds calcium and forms ion-permeable transmembrane channels in the CM, leading to alterations in membrane potential and subsequent cell rupture [133]. The *mprF* gene encodes PG lysyl-transferase. Mutations in *mprF* occur at high frequency in *S. aureus*, followed by *E. faecium*. Gain-of-function non-synonymous mutations in *mprF* increase the production of positively charged Lys-PG. Translocation of Lys-PG to the outer leaflet of the CM increases the net positive surface charge and reduces DAP binding (Figure 2b) [134,135]. A mutation in the *cls* gene, which reduces the negative charge of the CM, is the second most common mechanism conferring DAP resistance in *S. aureus* and MRSA. It typically acts synergistically with *mprF* mutations to collectively lead to significant resistance [136,137]. The *rpoB* and *rpoC* genes mediate resistance through a “global regulatory” mechanism. They encode the β and β’ subunits of RNA polymerase, core components of the bacterial transcription machinery [138]. Unlike *mprF* mutations, which often emerge relatively early under selective pressure, *rpoB* and *rpoC* mutations tend to appear later [139]. These global alterations enable bacteria to enter a new physiological state, with the common final adaptive pathway typically involving changes in the composition and properties of the CM to counteract antibiotic stress. For example, single point mutations in *rpoB*, such as A477D or A621E, can reduce susceptibility to both DAP and vancomycin [140,141].

Polymyxin kills bacteria by binding to LPS on the bacterial OM, disrupting membrane integrity [86]. Membrane modifications conferring polymyxin resistance are mediated by *PmrAB*/*PhoPQ* and *mcr-1*. *PhoP/Q* and *PmrA/B* are two of the most extensively studied two-component systems regulating OM composition in G^−^ bacteria [142]. Encoded chromosomally, these systems act as bacterial “sentinels” for sensing environmental stress [143]. Mutations in *pmrA/pmrB* or *phoP/phoQ* can lead to constitutive activation of LPS-modifying enzymes. These enzymes add Phosphatidylethanolamine (PEA) or 4-amino-4-deoxy-L-arabinose to LPS molecules, modifications that reduce polymyxin affinity for LPS and result in low-level resistance [144]. The *mcr-1* gene is an “acquired” resistance determinant obtained via mobile genetic elements such as plasmids [143]. It encodes a PEA transferase functionally similar to enzymes downstream of the *PmrAB/PhoPQ* systems. Since this modification does not require specific environmental stimulation, bacteria constitutively produce the MCR-1 enzyme, continuously modifying their LPS [145]. The emergence of *mcr-1* likely resulted from the capture of chromosomal genes, such as the inherent *pmrC* (which encodes a PEA transferase), onto plasmids, freeing them from their native complex regulatory constraints.

To combat resistance caused by *mprF*/*mcr-1*-mediated membrane charge alterations, research should focus on developing DAP analogs with reduced reliance on membrane electrostatic potential. Biphenyl-modified fatty acyl chains, for example, enable effective membrane insertion even with diminished negative charge, bypassing *mcr-1*-mediated resistance [76,77]. For multi-target DAP resistance, tailored multi-targeting antimicrobial LPs are needed. The representative compound Bac-51, featuring a hydrophobic trifluoromethyl biphenyl group at Orn7, enhances lipid II binding while improving membrane permeability and causing depolarization. This multi-mechanism approach boosts its potency against MRSA, Vancomycin-Resistant *Enterococcus* (VRE), and DAP-resistant strains by 8–256-fold compared to native bacitracin [146].

#### 3.1.2. Membrane Rigidification Approaches to Overcome Resistance

LPs initially bind to negatively charged CMs via electrostatic interactions, after which their hydrophobic tails insert into the hydrophobic core of the lipid bilayer to disrupt membrane integrity and form pores, ultimately leading to membrane depolarization and disintegration. However, straight-chain saturated FAs produced by the bacterial Type II FA Synthesis system increase membrane packing density, thereby inhibiting the membrane insertion of DAP [147]. On the other hand, functional impairment or mutation of the *pgpA* gene, which encodes PG phosphatase, reduces PG content in the membrane and alters membrane fluidity, consequently weakening the binding capacity of DAP [136,148,149]. To address DAP resistance resulting from reduced membrane fluidity, strategic modification of LP structures to enhance penetration through dense membrane structures and optimize interactions with membrane components has demonstrated considerable promise. For instance, incorporation of elongated lipid chains or fluorine atom substitution enhances hydrophobicity and oleophobicity, significantly improving membrane permeability and antibacterial efficacy. The fluorinated analog R6F exemplifies this approach, effectively disrupting membrane integrity and bacterial metabolism [150]. Biomimetic design strategies have yielded LP3K, an inverse-conical LP that exploits geometric antagonism with bacterial membrane lipids to efficiently perturb membrane integrity [151]. Furthermore, liposome-encapsulated formulations substantially improve LP retention and enhance anti-biofilm activity [152]. These findings underscore the importance of identifying novel functional groups that augment hydrophobic interactions with membranes and induce geometric antagonism, providing crucial insights for developing next-generation LPs against MDR pathogens.

### 3.2. Efflux Pumps Activation Approaches to Overcome Resistance

**Figure 3:**
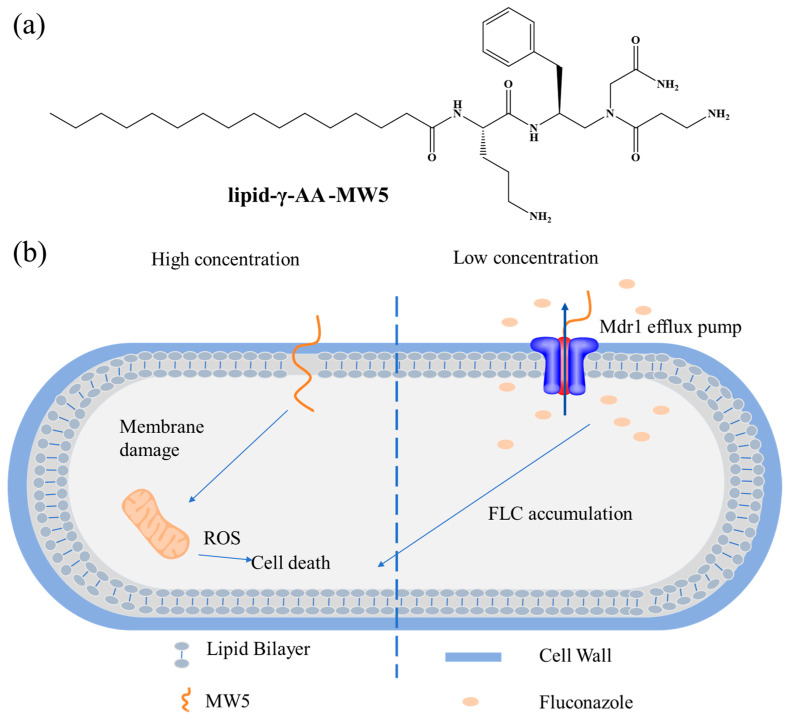
The design and antifungal mechanism of lipid-γ-AA peptides MW5. (**a**) The structure of MW5; (**b**) Proposed model for the antifungal activity of MW5, alone and synergistic with fluconazole (FLC). MW5 targets the plasma membrane, causing permeabilization and, at high levels, ROS-induced cell death. At low concentrations, it inhibits the Mdr1 efflux pump, enhancing FLC accumulation and overcoming efflux-mediated resistance [160].

For intracellularly acting LPs, such as C10-PR-Spn [153], and other antibiotics, microorganisms activate multiple defense mechanisms, including efflux pump systems, to ensure survival [153,154,155]. Specifically, G^+^ bacteria upregulate membrane repair genes and efflux pump expression via the LiaFSR signaling pathway; G^−^ bacteria utilize the AcrAB-TolC tripartite efflux complex to expel antimicrobial agents; and *Candida albicans* overexpresses MDR/CDR family efflux proteins, such as P-gp, to reduce intracellular drug accumulation, thereby establishing a resistant phenotype [156,157,158]. Notably, certain LPs demonstrate the ability to counteract such efflux pump-mediated resistance. For instance, C12(ω7)K-β12 induces transient membrane depolarization, disrupting the proton motive force-dependent efflux process and resensitizing bacteria to intracellularly targeted antibiotics [159]. Concurrently, the lipidated γ-AApeptide derivative MW5 directly inhibits the function of the fungal multidrug efflux protein MDR-1, increasing intracellular fluconazole concentration and reversing azole resistance (Figure 3) [160]. These findings may inform the combined use of efflux pump-modulating LPs with conventional antibiotics. Furthermore, in-depth analysis of the structure–activity relationships of LPs could provide crucial lead compound structures for developing novel inhibitors specifically targeting efflux pumps.

### 3.3. Cell Wall Thickening Approaches to Overcome Resistance

The CW serves as a critical extracellular structure that indirectly modulates the antibacterial activity of LPs by altering membrane tension and stress states. Key CW modification systems implicated in LP resistance include the *WalKR* two-component system and the *dltABCD* operon. Mutations in *WalKR*, which regulates CW hydrolase activity, disrupt CW metabolic homeostasis, leading to wall thickening that delays or prevents LP diffusion to membrane targets (Figure 2b) [161]. Additional studies demonstrate that *walR* mutation-induced wall thickening impedes extracellular transport of intracellular polysaccharides and proteins, thereby compromising biofilm formation capacity [162]. Furthermore, mutations in the *dltABCD* operon enhance D-alanylation of teichoic acids in G^+^ bacteria, increasing CW positive charge density and weakening electrostatic interactions with positively charged LPs [163]. To address bacterial resistance resulting from CW thickening, it is necessary to target the necessary bacterial vulnerabilities to bypass the drug resistance sites and utilize multi-target LPs. Representative examples include Bac-51 and BD-V-2, which simultaneously target both the CW precursor lipid II and the bacterial membrane, demonstrating effective activity against resistant strains [146,164].

### 3.4. Biofilm Formation Approaches to Overcome Resistance

Bacterial biofilm formation confers antibiotic resistance through mechanisms distinct from acquired genetic resistance. It primarily involves the establishment of a physical-physiological barrier, the development of metabolically inactive persister cells, and alterations in membrane potential [165,166,167]. The extracellular polymeric substance matrix of the biofilm physically blocks or adsorbs antibiotic molecules, severely impeding their diffusion and leading to subinhibitory concentrations in the biofilm core [165]. Bacteria within the biofilm often enter a slow-growing or dormant state as persisters, characterized by reduced membrane metabolic activity, which diminishes the efficacy of membrane-targeting drugs like LPs [166]. Furthermore, studies indicate that bacteria in the outer regions of the biofilm can adapt by modulating their metabolism and membrane potential. This adaptation, often involving a lowered membrane potential that sustains glucose consumption, slows the accumulation of antibiotics, providing a critical time window for bacterial adaptation and survival [167].

Current research strategies to address biofilm-mediated resistance primarily focus on monotherapy optimization, synergistic combinations, and targeted delivery. For instance, early studies demonstrated that DAP monotherapy exhibits superior bactericidal activity against biofilm-forming MRSA compared to vancomycin [168]. Conversely, the combination of the short LP Pal-Lys-Lys with vancomycin significantly enhanced the killing of biofilm-embedded bacteria both in vitro and in vivo, suggesting its potential to help other antibiotics penetrate the biofilm barrier [169]. Targeted delivery systems aim to achieve rapid bactericidal effects by elevating drug concentrations at the infection site. An example is the combined application of polymyxin B-loaded liposomes with ultrasound microbubbles, which nearly eradicated resistant *Acinetobacter baumannii* biofilms at a concentration as low as 2 µg/mL [170]. Furthermore, a bio-inspired delivery system, featuring cationic peptide-conjugated liposomes with a virus-like structure, demonstrated potent broad-spectrum antimicrobial activity, achieving killing rates of 100%, 98%, and 89% against *Pseudomonas aeruginosa*, *Escherichia coli*, and MRSA, respectively, within 10 h [171]. The core objective of these strategies is to overcome the physical barrier of biofilms and target the dormant bacteria within. Future development of integrated treatment regimens—through bio-inspired design, smart materials, and rational drug combinations—holds significant promise for effectively penetrating biofilms, eradicating persister cells, preventing recurrence, and ultimately translating these advances into clinical therapies for intractable biofilm-associated infections.

LP resistance involves a multi-layered defense: primary changes in membrane charge (e.g., via *MprF*/*mcr-1*) block LP binding, while secondary adaptations reduce membrane fluidity and alter cell wall structure to inhibit pore formation. Efflux pump activation further intensifies resistance. Systems such as ParRS integrate these mechanisms, driving high-level, cross-resistant phenotypes [172]. To counter this, designing allosteric or multi-target LPs that bypass common resistance pathways—coupled with systematic structure–activity analysis of existing potent analogs—can guide the development of next-generation therapies.

**Table 4:** Classification of Resistance Mechanisms in LPs.

Resistance to LP	Resistant Strain	Resistance Gene	Resistant Phenotype	References
DAP	*S. aureus* *E. faecium*	*MprF*	Increased Lys-PG synthesis. Elevated surface positive charge.	[134,135]
DAP	*S. aureus* MRSA	*Cls*	PG accumulation. Reduced CM negative charge.	[136,137]
DAP Microcin J25	*E. coli*	*RpoB/RpoC*	Delayed rpoB/C mutations (post-mprF). Bacterial physiological state shift.	[139,140,141]
Polymyxins	*E. coli* *Salmonella* *K. pneumoniae* *Enterobacter cloacae* *Proteus mirabilis* *A. baumannii*	*Mcr-1*	Constitutive LPS modification. Reduce the net negative charge on the surface of CMs.	[145]
Polymyxins	*E. coli* *Salmonella* *K. pneumoniae* *P. aeruginosa*	*PmrAB*/*PhoPQ* two-component systems	Modification of LPS with PEA or 4-amino-4-deoxy-L-arabinose.	[144]
DAP	Mycobacterium *P. aeruginosa*	Type II FA Synthesis system	Straight-chain saturated FA production. Increased membrane packing density.	[147]
DAP	*C. striatum* *S. aureus*	*PgsA*	Decreased membrane PG Disrupted membrane fluidity	[136,149]
DAP	*E. faecalis* *E. faecium;* *S. pneumoniae.*	*LiaFSR*	Expression of efflux pumps Upregulation of membrane repair genes	[156]
LP	*E. coli* *K. pneumoniae*	*AcrAB-TolC*	**AcrB**: Substrate capture and conformational change **AcrA-TolC**: Channel formation across the envelope **Complex**: Drug export and virulence enhancement	[157]
PMB Colistin	*C. albicans* *L. lactis*	P-glycoprotein *LmrA* protein	MDR/CDR (P-glycoprotein/*LmrA* protein) mediated efflux	[158,173]
DAP Vancomycin	Coagulase-negative *staphylococci*	The *WalKR* two-component system	CW thickening	[161,162]
DAP	*S. aureus* *E. faecalis* *L. monocytogenes* *S. pyogenes*	*DltABCD* operon	Increased CW positive charge	[163]
PMB Colistin	*P. aeruginosa*	*ParRS* two-component system	LPS modification Enhanced efflux Porin downregulation	[172]

## 4. Structural Optimization of LPs for Enhanced Functionality

The pharmacological activity of LPs is determined by their core structure—a peptide backbone linked to a lipid tail. This review begins by discussing three key rational design strategies: (1) fine-tuning the peptide backbone through amino acid substitution and side-chain modification; (2) optimizing the lipid chain (length, branching, saturation) and introducing structural variations to overcome resistance and improve selectivity; and (3) leveraging the self-assembling properties of LPs to develop advanced delivery systems that enhance targeting and reduce toxicity.

### 4.1. Structural Optimization of the Peptide Scaffold

Analysis of established LP scaffolds shows that their peptide backbones often incorporate aliphatic/aromatic, D-type, or non-canonical amino acids, along with side-chain modifications, to fine-tune hydrophobicity, charge, and metabolic stability. Furthermore, β-Peptide scaffolds further offer enhanced protease resistance compared to α-peptide frameworks.

#### 4.1.1. Introduction of Specific Amino Acids

The biological activity and toxicity of LPs are fundamentally governed by their precise amphipathic architecture. The amino acid composition and sequence of the peptide backbone critically dictate the delicate balance between antimicrobial potency, membrane selectivity, and toxicological risk. Key determinants include the spatial distribution of hydrophobic residues, the incorporation of rigid motifs, the proportion of polar amino acids, and, most notably, the net positive charge.

**Figure 4:**
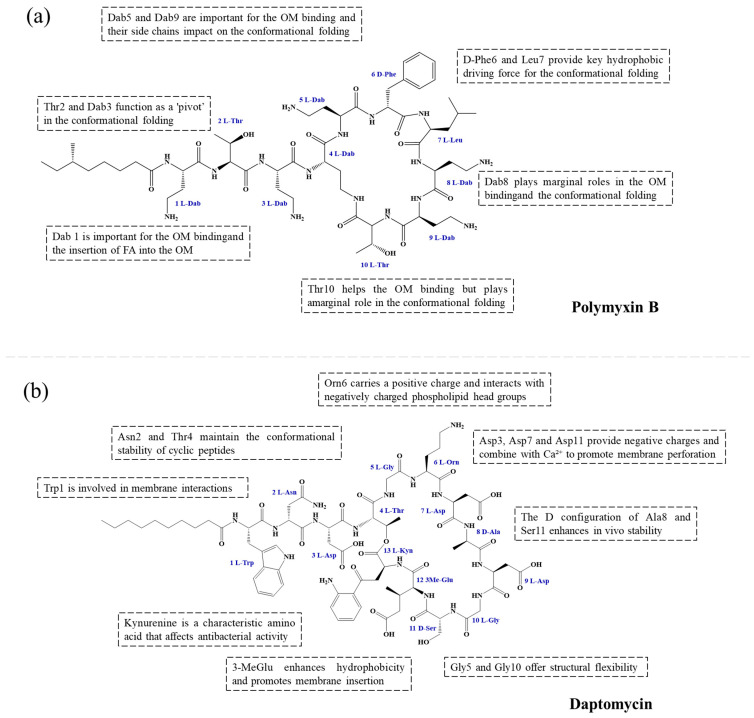
The SARs of representative LPs-PMB and DAP [122]. (**a**) The SARs of PMB. (**b**) The SARs of DAP (The PMB and DAP structure was drawn by ChemDraw 20.0.).

Given that hydrophobicity is primarily contributed by the lipid tail, hydrophobic amino acids within the peptide scaffold are generally limited. As illustrated in the accompanying table, non-polar aliphatic residues (Leu, Ile, Gly, Ala) typically number between 2 and 4, often located within cyclic structures, where they fine-tune amphipathicity and conformational flexibility. Aromatic residues (Trp, Phe) enhance membrane-disruptive capacity via combined hydrophobic and π-π stacking interactions, albeit with a potential increase in toxicity. For instance, the substitution of 6D-Phe in PMB with 6D-Leu to yield polymyxin E (colistin) results in subtle structural differences, contributing to PMB’s stronger antimicrobial activity and polymyxin E’s relatively lower nephrotoxicity (Figure 4a). The introduction of rigid proline residues can segregate hydrophilic and hydrophobic domains, preventing excessive self-aggregation and thereby reducing hemolytic toxicity [174]. Studies on Figainin-2PL and temporin-PE demonstrate that replacing proline with alanine or tyrosine increases hemolytic activity by 10- and 13-fold, respectively [175,176].

A balanced proportion of polar amino acids is essential for optimal amphipathicity. DAP, with polar residues constituting 58.3% of its scaffold, exhibits a favorable safety profile; however, its limited cationic residues necessitate Ca^2+^ binding to supply the positive charge required for membrane interaction (Figure 4b). In contrast, polymyxins feature a high density of cationic Dab residues, which are crucial for electrostatic interaction with the negatively charged LPSs of G^−^ bacterial membranes. Each additional positive charge can increase the binding constant to bacterial PG and CL by 10- to 100-fold [177]. Unfortunately, this high net positive charge also drives non-specific binding to mammalian membranes, underlying the nephro- and neurotoxicity associated with polymyxins [178]. Nevertheless, scaffolds composed predominantly of cationic residues (Lys, Arg) hold promise for non-systemic applications, such as antimicrobial biomaterials. For example, short cationic LPs have demonstrated complete eradication of G^+^ bacterial biofilms on surfaces at concentrations as low as 64 μg/mL [179].

In summary, structural analyses of classic LPs like polymyxins and DAP underscore that optimizing the balance between amphipathicity and cationic charge is paramount for designing potent yet low-toxicity antimicrobial agents. Concurrently, designs with higher charge density may offer unique advantages for specific ex vivo applications, such as coating of biomedical devices.

#### 4.1.2. Modification of Amino Acids

Modification of the amino acid moiety in LPs encompasses both conformational alterations and side-chain functionalization. Conformational engineering, such as substituting L- with D-amino acids or replacing α- with β-amino acids, enhances the overall structural stability of the LP scaffold.

**Figure 5:**
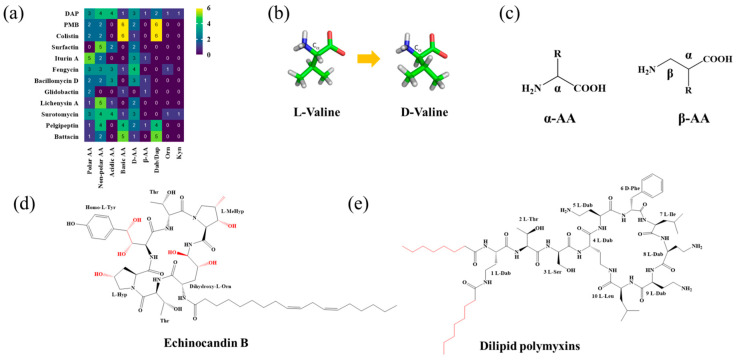
Structural Optimization of the Peptide Scaffold. (**a**) The distribution of Polar AA, Non-polar AA, Acidic AA, Basic AA, and Uncommon AA (including D-AA, β-AA, Dab, Dap, Orn, Kyn) in representative LPs. (**b**) The transformation from L-AA to D-AA. (**c**) α-AA and β-AA. (**d**) The structure of Echinocandin B (the red part represents hydroxyl modification). (**e**) The structure of Dilipid Polymyxins (the red part represents the acyl modification).

D-Amino acids, although rare in higher organisms and primarily involved in bacterial polypeptide synthesis, are often strategically incorporated at key positions—such as within cyclic structures or functional β-turn motifs—in LPs. Their inclusion represents a powerful and versatile strategy for optimizing LP therapeutics. Systematically, D-amino acids improve protease resistance, prolong plasma half-life, enhance biofilm penetration, and help reconcile antimicrobial potency with host cytotoxicity (Figure 5b). For instance, brevibacillin 2V, linear analog 22, and the ultrashort LP C12-RRW-NH_2_, each containing multiple D-residues, exhibit remarkable stability against proteolytic degradation [73,180,181]. Furthermore, D-amino acid substitutions can effectively decouple antibacterial activity from hemolytic or cytotoxic effects. In LPs BP475 and BP485, the presence of D-Phe at position 4 maintains potent antimicrobial activity (MIC 0.8–6.2 μM) while significantly reducing hemolysis [182]. Similarly, brevibacillin 2V, which contains noncanonical and D-amino acids, displays strong antibacterial efficacy with markedly lower hemolytic and cytotoxic profiles compared to conventional analogs [180]. The impact of D-amino acids on bioactivity can also stem from their ability to disrupt regular secondary structures. NMR analysis of BP475 revealed that D-Phe at position 4 breaks the α-helical conformation, inducing a partially folded helical structure associated with high antimicrobial activity and low hemolysis [182]. Notably, D-amino acids are intrinsic features of several naturally occurring LPs with unique activities, such as gausemycins A and B, where they occupy unusual positions [183], and they are recognized as effective tools for tuning the therapeutic properties of AMPs [184]. Rational design based on natural templates, such as Brevicidine analogs, further validates the value of D-amino acids in achieving broad-spectrum activity, high stability, and low propensity for resistance induction [181]. Future research will continue to leverage advanced stereochemical analysis [185], rational design principles [181,182], and natural product-inspired modification [180,183], to fully exploit the potential of D-amino acids in developing next-generation LPs with enhanced stability, reduced toxicity, and potent activity against MDR pathogens.

Compared to traditional α-peptides such as polymyxins, β-peptides exhibit superior metabolic stability and high resistance to protease hydrolysis (Figure 5c). For instance, a novel tapered block co-β-peptide achieves potent and selective killing of G^−^ bacteria through specific backbone interactions with LPS, demonstrating effective biofilm eradication and low cytotoxicity in murine models [186]. Similarly, incorporating β-alanine into short cationic antimicrobial LPs enhances their propensity for protofibril formation, which improves stability and activity against *Staphylococcus epidermidis* and *Candida albicans* while reducing cytotoxicity toward human keratinocytes [187]. Furthermore, introducing pharmacophoric groups onto amino acid side chains within the peptide scaffold can significantly enhance antibacterial efficacy. Fluorination at the 5- or 6-position of the tryptophan side chain, for example, doubles antimicrobial activity without increasing hemolytic toxicity [188]. The hexapeptide core of the echinocandin family LP Echinocandin B is entirely composed of L-homotyrosine, L-dihydroxyornithine, L-hydroxyproline, L-methylhydroxyproline, and two L-threonine residues. These amino acids all bear polar modification groups such as hydroxyl groups, and these modifications determine their unique antifungal mechanism of non-competitively inhibiting β-1,3-glucan synthase [189]. Internal lipidation represents another promising strategy for developing selective LPs. Introducing a secondary acyl chain at the side chain of the L-Dab residue at position 1 of polymyxin creates a dilipidated analog that not only confers new activity against G^+^ bacteria but also evades the MexAB-OprM efflux pump and exhibits adjuvant activity similar to PMBN [190]. Alternative lipidation via side-chain acylation of lysine residues—rather than conventional N-terminal modification—with octanoic (C_8_) or decanoic (C_10_) acid in peptides such as LL-I, LK6, and ATRA-1 also enhances antimicrobial activity while reducing cytotoxicity [191]. However, not all modifications yield uniformly improved profiles. For instance, substituting the R6 or R7 residue of PMB with more lipophilic d/l-2-aminocanonic acid or a biphenylcarbamoyl group enhances activity against resistant strains (MIC < 32 μg/mL) but concurrently increases cytotoxicity toward HepG2 cells [192].

These findings collectively indicate that the effectiveness of side-chain pharmacophore modifications is highly context-dependent, influenced by the original peptide sequence and structure. Optimal modification strategies—including the choice of functional group, lipid chain length, and site of incorporation—require careful and systematic evaluation.

### 4.2. Optimization of FA Tailoring

The N-terminal lipoyl group of LPs critically modulates antimicrobial activity by adjusting molecular hydrophobicity. Optimizing its structure—type, length, branching, saturation, and chemical modification—is therefore essential for balancing antibacterial potency and host–cell selectivity.

**Figure 6:**
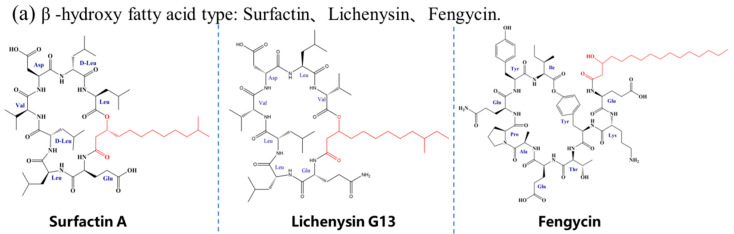

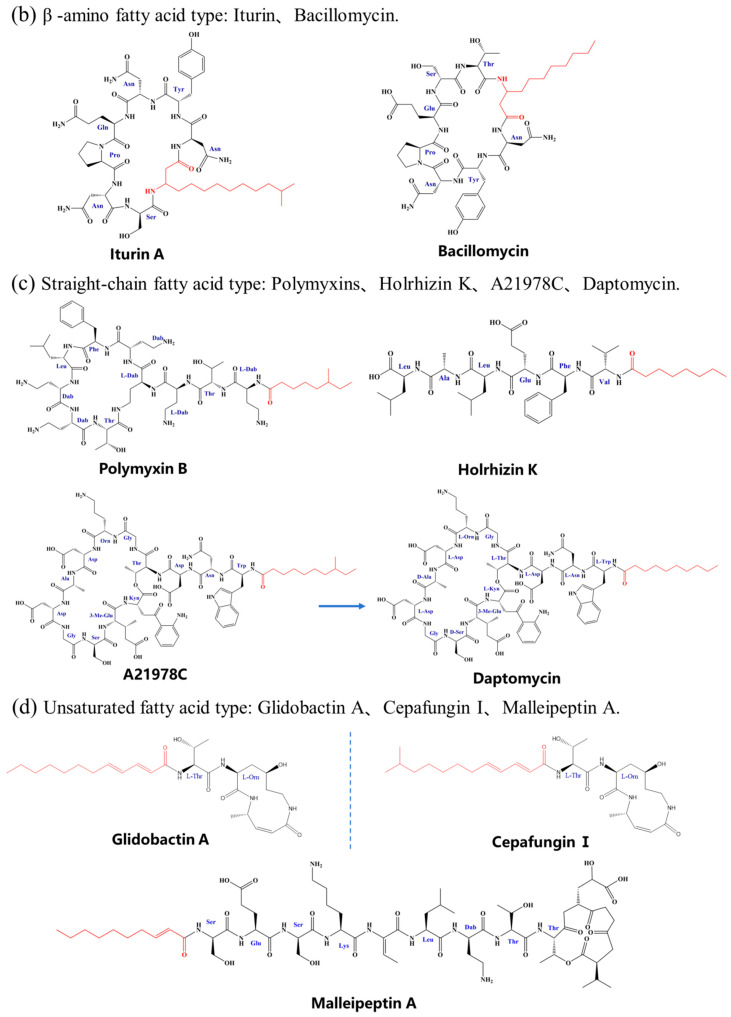

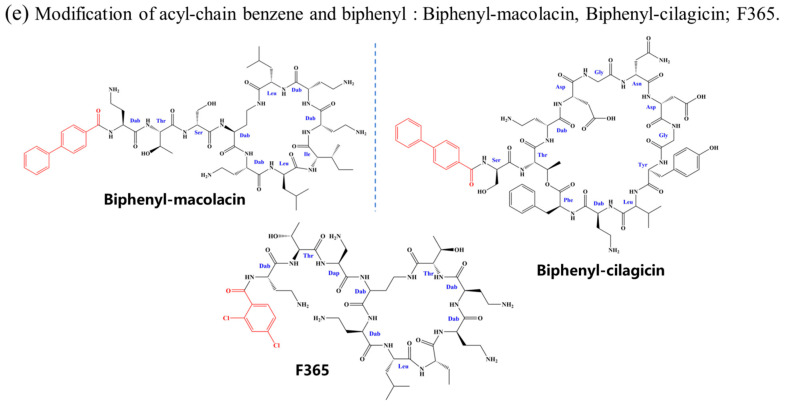
Classification of LP structures by FA types. (**a**) β-hydroxy FA type: Surfactin, Lichenysin, Fengycin. (**b**) β-amino FA type: Iturin, Bacillomycin. (**c**) Straight-chain FA type: Polymyxins, Holrhizin K, A21978C, DAP. (**d**) Unsaturated FA type: Glidobactin A, Cepafungin I, Malleipeptin A. (**e**) Modification of acyl-chain benzene and biphenyl: Biphenyl-macolacin, Biphenyl-cilagicin; F365. (The LP structure was drawn by ChemDraw 20.0. The structural differences in the acyl chains of representative LPs in the figure are indicated in red).

LPs can be classified based on the structure of their FA moieties, which include β-hydroxy FAs, β-amino FAs, linear or branched saturated FAs, and unsaturated FAs (Figure 6). The hydroxyl group in β-hydroxy FAs enhances molecular hydrophilicity and overall amphipathicity. For instance, in surfactin, the β-hydroxy FA cooperates with its heptapeptide ring to efficiently penetrate *S. aureus* biofilms, resembling a “molecular drill” mechanism [193]. β-Amino FAs alter charge distribution and chemical reactivity due to the presence of an amino group. In cationic LPs such as iturin and bacillomycin, this amino group, along with positively charged residues in the peptide chain, contributes to the overall positive charge [91,128].

The length of the fatty acyl chain significantly influences LP activity and selectivity. Medium-chain lengths (C8–C12) generally optimize the amphipathic balance and confer strong antibacterial activity. A representative example is DAP, which shows peak antimicrobial activity when linked to a decanoyl (C10) chain [194,195]. Longer chains (C13–C21) tend to enhance interactions with eukaryotic membranes. For example, fatty-acylated melittin analogs exhibit improved antifungal activity, and conjugation of a long-chain FA to R-Lycosin-I significantly enhances its anticancer efficacy [196,197].

Branched FA structures further modulate LP function. The branched chain of surfactin disrupts membrane integrity via a “wedge-insertion” model and lowers the critical micelle concentration, thereby improving surface activity at low concentrations [198]. Terminal methyl branching in iturin A, mycosubtilin, and bacillomycin L increases hydrophobicity [199]. In PMB, terminal methyl branching facilitates selective insertion into bacterial membranes containing specific “impurity lipids” such as 1-Palmitoyl-2-oleoyl-sn-glycero-3-phosphoethanolamine, 1-Palmitoyl-2-oleoyl-sn-glycero-3-phospho-rac-(1-glycerol), or lipid A [200].

Saturated fatty acyl chains, owing to their structural regularity, readily align with saturated phospholipids in bacterial membranes, promoting the formation of “barrel-stave” type transmembrane channels. For instance, a decanoyl chain can significantly enhance the activity of polymyxin against *mcr-1*-positive bacteria [201]. Although less common, unsaturated fatty acyl chains are found in natural LPs such as glidobactin A, cepafungin I, and malleipeptin A (Figure 5d), all of which exhibit antitumor activity [202,203,204]. The flexibility of unsaturated chains may improve penetration into highly fluid eukaryotic membranes while potentially reducing membrane-disruptive effects on mammalian cells.

In addition to the branched structures formed by terminal methylation mentioned earlier, various other modifications at the fatty acyl terminus have been explored, including glucosylation, biphenyl incorporation, and the introduction of hydroxyl, carboxyl, amino groups, or disulfide bonds. For example, glucosylation of R-lycosin-I enhances its tumor-targeting capacity [205]. Biphenyl modifications, as seen in macolacin and cilagicin, engage in strong hydrophobic interactions and π-π stacking with the LPS layer of the bacterial OM, effectively overcoming *mcr-1*-mediated resistance (Figure 5e) [76,77]. The incorporation of a reduction-sensitive disulfide bond into the lipid tail of polymyxin analogs has yielded derivatives that retain antibacterial activity comparable to PMB while significantly reducing toxicity toward human renal proximal tubular epithelial cells [43]. Furthermore, substitutions such as oxidation, chlorination, or hydroxylation at the fatty acyl base can mitigate cytotoxicity. In LPs such as puwainaphycins and minutissamides—which typically exhibit both antifungal activity and cytotoxicity—oxidized variants show a reduced cytotoxic impact compared to unsubstituted counterparts. Hydroxylation of the fatty acyl base further diminishes or even abolishes cytotoxicity, whereas the activity of chlorinated analogs varies depending on the position of the chlorine atom along the FA chain [206].

### 4.3. Construction of Novel Delivery Systems for LPs

Despite promising in vitro activity, the in vivo efficacy of naked LPs is often limited by enzymatic degradation, poor bioavailability, and off-target toxicity. Targeted delivery systems—including LP self-assembly, surface functionalization, localized in situ synthesis, and material synergy—offer strategies to enable more precise and effective in vivo delivery.

**Figure 7:**
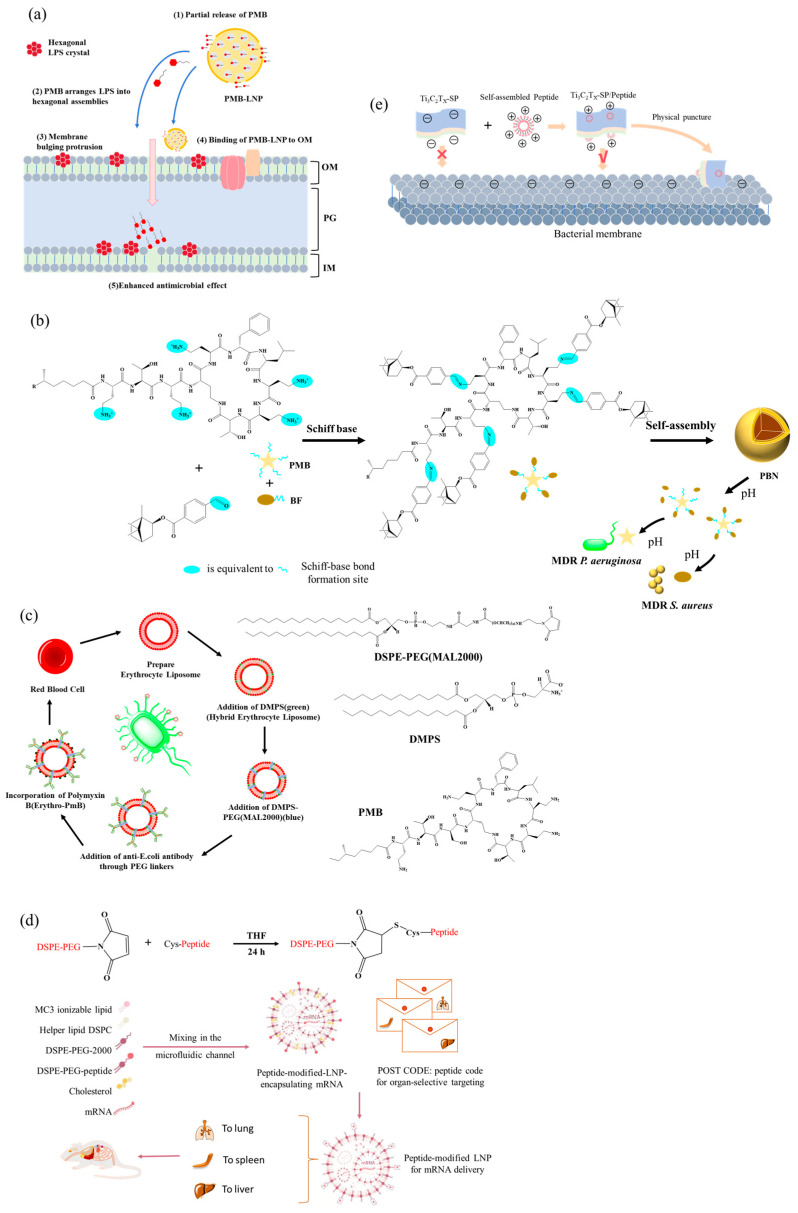
Targeted delivery technology of LPs. (**a**) LNP-PMB encapsulation [210], (**b**) self-assembly [211], (**c**) surface functionalization [212], (**d**) localized in situ synthesis [19,21,213], and (**e**) material synergies [214].

The amphiphilic nature of LPs drives their self-assembly into diverse nanostructures such as micelles, vesicles, nanotubes, fibers, or nanobelts, facilitating their rapid development as therapeutic agents with antibacterial, antifungal, antiviral, or anticancer activities [207]. Recent studies demonstrate that polymyxin can self-assemble with (E)-2-heptenal in aqueous solution, effectively shielding its positive charge, reducing cytotoxicity, reversing resistance in clinical strains, and efficiently preventing and eradicating biofilms [208]. Leveraging gastrointestinal pH gradients, co-delivery systems comprising oppositely charged nanoparticles (formaldehyde-treated and alginate-coated nanoparticles) can electrostatically deliver niclosamide and colistin. Such systems transform into loose structures in the neutral intestinal environment, enabling sustained drug release, alleviating inflammation and intestinal damage caused by bacterial infection, and offering good biocompatibility and palatability [209]. Encapsulation of cationic PMB within anionic lipid nanoparticles (LNPs) via electrostatic interactions prolongs circulation, enables liver-targeted accumulation, and exhibits superior anti-G^−^ activity and tolerance both in vitro and in vivo compared to free PMB (Figure 7a) [210]. Furthermore, carrier-free nanodrugs constructed via dynamic Schiff base bonds exhibit pH-responsive behavior in the acidic microenvironment of infection sites, enabling simultaneous release of PMB and bornyl p-aldehyde benzoate to, respectively, target MDR *P. aeruginosa* and *S. aureus*. This approach effectively clears mixed bacterial infections and accelerates wound healing in vivo (Figure 7b) [211]. Surface functionalization further enhances the precision targeting of LP-based nanodelivery systems. For instance, antibody-modified hybrid erythrocyte liposomes enable targeted delivery of PMB to specific pathogens, significantly improving targeting efficiency while reducing nephrotoxicity (Figure 7c) [212]. Surface engineering of LNPs by integrating natural amino acids or functional molecules enables organ-specific mRNA delivery to the lungs, liver, spleen, and other organs, paving the way for localized in situ synthesis of LPs to minimize systemic exposure and associated side effects (Figure 7d) [19,21,213]. Synergistic effects can also be achieved by combining LPs with functional materials. For example, the self-assembled system of narrow-spectrum PMB and bornyl p-aldehyde benzoate shows superior antibacterial efficacy compared to their simple combination [211]. LP–MXene nanosheet composites combine physical membrane disruption and membrane-anchoring functions, demonstrating broad-spectrum and potent antibacterial activity (Figure 7e) [214]. Colistin–succinyl chitosan conjugates maintain antibacterial activity while reducing nephrotoxicity through sustained release [215]. Moreover, conjugating the siderophore fimsbactin with DAP exploits bacterial iron-uptake systems for “Trojan horse” delivery, extending the use of this G^+^-targeting antibiotic to treat MDR G^−^ bacterial infections [216].

LP delivery systems are evolving from simple carriers into multifunctional platforms. Integration with nanotechnology through strategies such as encapsulation and conjugation enhances their stability, targeting, and safety. Future development will focus on applying rational design to existing self-assembly approaches, addressing challenges in scalability and biosafety, to enable effective and low-toxicity nanoformulations for anti-infection therapy, gene delivery, oncology, and other major disease areas.

In summary, rational LP design relies on synergistically optimizing the peptide and lipid components together, not independently. A “functional decoupling” strategy assigns bacterial surface recognition to the hydrophilic peptide and membrane insertion to the hydrophobic lipid tail, while fine-tuning the overall hydrophilic-lipophilic balance to minimize aggregation and toxicity. These parameters can be iteratively refined by integrating computational modeling with combinatorial library screening, facilitating the discovery of novel LPs with enhanced antimicrobial activity, lower toxicity, and strong efficacy. against drug-resistant pathogens.

## 5. Emerging Approaches and Enabling Technologies for LP Design and Discovery

A survey of the literature from the past 5–10 years highlights several pivotal and emerging methodologies that are expanding the frontiers of LP design and discovery. These approaches can be broadly categorized into two complementary strands: (1) the re-engineering of biosynthetic machinery, exemplified by NRPS engineering; and (2) computational and synthetic strategies, including HTS platforms, the syn-BNPs paradigm, and AI-driven de novo design. Collectively, these technologies are shifting the field from traditional, labor-intensive modification towards a more rational, predictive, and high-throughput paradigm for generating novel LP therapeutics with tailored properties.

### 5.1. NRPS Engineering

**Figure 8:**
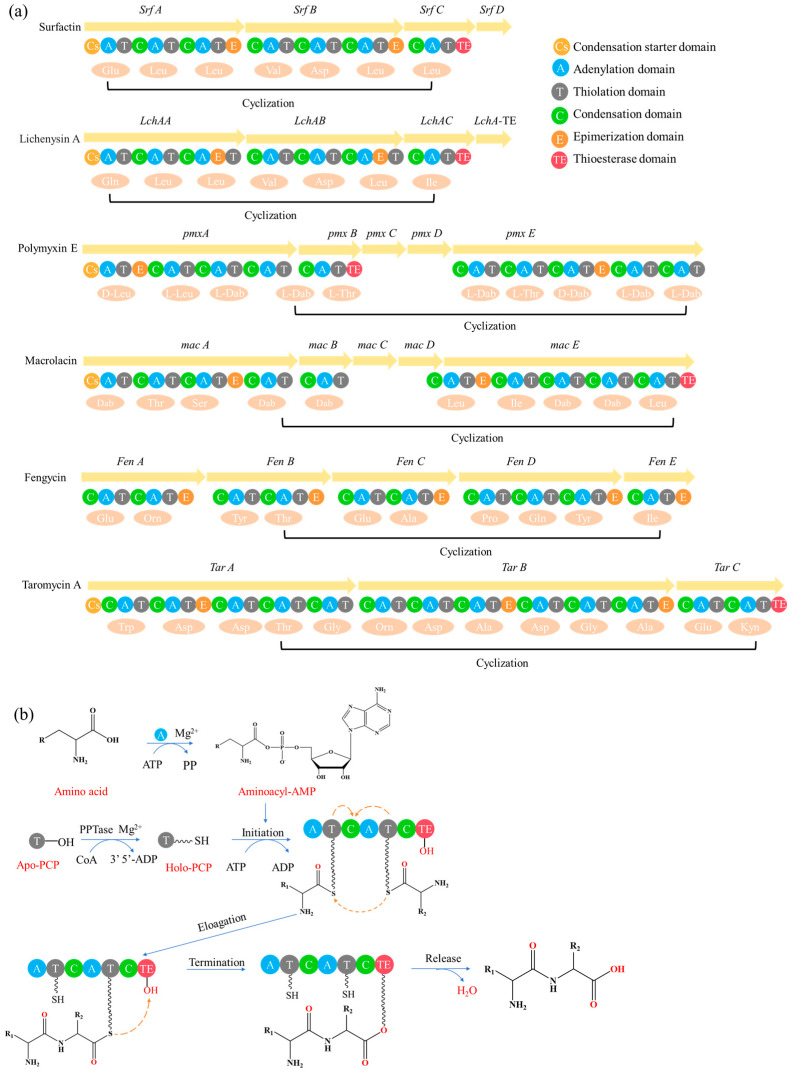
Biosynthesis of representative LPs by NRPS assembly lines. (**a**) Architecture of representative NRPS gene clusters for LPs. Key catalytic domains are color-coded: starter condensation (Cs) domain (yellow); adenylation (A) domain (blue); thiolation (T) domain (also known as peptidyl carrier protein (PCP); gray); condensation (C) domain (green); epimerization (E) domain (orange); and thioesterase (TE) domain (red-brown). (**b**) The NRPS mechanism for LP synthesis. The process involves initiation, elongation, and termination stages. During initiation, the A domain activates a specific amino acid using ATP to form an aminoacyl-adenylate (aminoacyl-AMP) intermediate. The 4′-phosphopantetheine arm of the holo-T domain (the active form) then performs a nucleophilic attack on this intermediate, loading the amino acid onto the T domain to form a covalent aminoacyl-S-T (AA-S-PCP) thioester linkage, with the concomitant release of AMP. This is followed by elongation, where the growing peptide chain is transferred to the next module for additional rounds of condensation, and finally termination, where the full-length peptide is released, often through macrocyclization or hydrolysis catalyzed by the TE domain.

**Figure 9:**
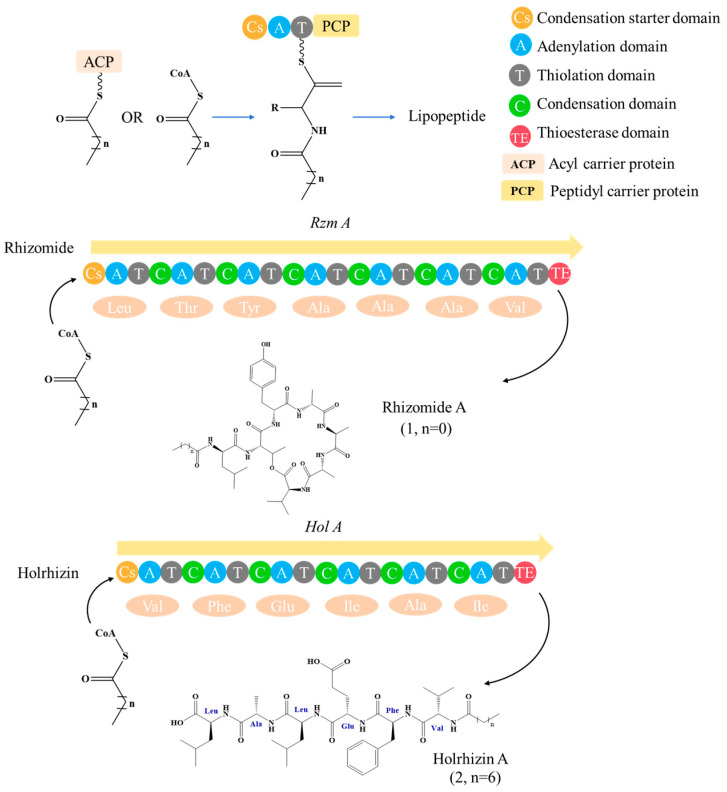
Biosynthesis of the FA moiety in LP BGC. (Take the partial synthesis process of LPs of Rhizomide and Holrhizin as an example [230]).

The modular “assembly line” mechanism of NRPS provides an efficient platform for the rational design and diversified synthesis of LPs (Figure 8) [217]. Its core engineering strategy focuses on the precise reprogramming and integration of specific functional domains: Adenylation (A) domain engineering combined with CRISPR-Cas9 technology can achieve the customization of amino acid units by replacing the key residues that determine substrate specificity or the conserved flavin-redoxin subdomain, and has been successfully applied in the preparation of novel high-yield surfactant analogs and diketopiperazine, etc. [74,218,219,220,221]. Thiesterase (TE) domain engineering can effectively regulate the cyclization, release, and yield of products by manipulating the position and type of the TE domain [222,223,224,225,226,227,228]. Examples show that by disrupting competitive pathways and overexpressing medium-chain acyl-ACP-thiesterase, the yield of surfactants can be increased by 34% [227]. Initiation condensation (Cs) domain engineering can rationally alter the FA chain length and properties of LPs by site-specific mutagenesis or domain exchange of key residues in the substrate binding pocket. It has been successfully applied to the derivatization of multiple family LPs, such as iturin and rhizomide (Figure 9) [128,229,230,231]. For instance, site-directed mutagenesis was carried out on the key residues (F208/K287/A332) of the substrate binding pocket in the Cs domain of the iturin family LPs, successfully obtaining novel analogs with extended lipid chains [128]. In addition, Module Swapping is another key strategy for diversifying LP structures. For instance, replacing specific modules in surfactants or DAP synthases can efficiently generate analogs with new amino acid sequences while maintaining activity [57,232,233,234]. Beyond the modification of NRPS themselves, the introduction of customized enzymes such as acyltransferases (like GdvG) can also achieve the synthesis of innovative structures such as polyketone-ribosomal peptide hybrid LP- Goadvionin A4 [234]. Meanwhile, “clipping enzyme” strategies such as glycosylation, halogenation, hydroxylation, and complex modifications catalyzed by P450 enzymes can further enrich the chemical diversity and biological activity of LPs. For instance, glycosylation enhances the selectivity of the anti-cancer peptide R-lycosin-I, and halogenation strengthens the activity of ramoplanin [235,236,237,238,239,240,241]. To achieve the efficient mining of the above-mentioned engineered LPs, the HTS technology driven by synthetic biology combines NRPS engineering with microfluidics, fluorescence sensing, etc., to construct a closed loop of “design-build-test-learn”, which can conduct parallel analysis of thousands of variants, thereby rapidly identifying high-performance targets from a large library and greatly accelerating the discovery process of lead LPs.

### 5.2. HTS Driven by Synthetic Biology

**Figure 10:**
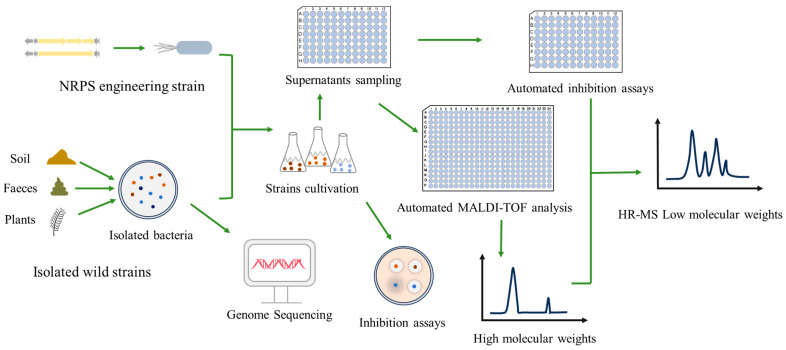
The HTS process is driven by synthetic biology [245]. The process encompasses three major stages: Library Construction, involving the design and cloning of a diverse NRPS gene library into a production host; Primary Screening, where the library is evaluated for antimicrobial activity on agar plates; and Advanced Screening and Metabolite Profiling, an automated phase where high-throughput bioactivity assays are integrated with high-resolution mass spectrometry to characterize the LP metabolites from validated hits.

At present, HTS techniques have achieved HTS and functional analysis of LP synthesis strains. For example, in the microfluidic platform, the sensing liposomes loaded with calcium yellow green have successfully achieved reliable detection of brevistin S synthesis, increasing the enrichment efficiency of peptide-producing strains by 14.5 times [242]. Semi-quantitative strain screening based on polydiacetylene vesicles and the precise concentration determination system of bromothymol blue/cetylpyridine chloride can achieve rapid analysis [243,244]. Automated sensitive detection was achieved through the fluorescence quenching platform, and downstream verification was carried out by Reversed-Phase Ultra-Performance Liquid Chromatography (RP-UPLC) and Matrix-Assisted Laser Desorption/Ionization Time-of-Flight Mass Spectrometry (MALDI-TOF MS) (Figure 10) [245]. In the functional screening, the strategy based on Real-Time Polymerase Chain Reaction (RT-PCR) successfully identified *B. amyloliquefaciens* 7D3, which produced LPs- surfactins and plipastatins that could disrupt the membrane integrity of *Fusarium gramineum* and inhibit spore germination [246,247]. In the future, integrating NRPS engineering with HTS technology is expected to build a closed-loop optimization system of “design-build-test-learn”. This collaborative strategy will significantly accelerate the discovery and optimization process of new LPs. This process can construct a LP variant library through NRPS engineering or heterologous expression of gene clusters, and then rapidly evaluate the LP yield, structure, and biological activity at the nanoscale by using microfluidic droplet encapsulation, colorimetric sensing, fluorescence detection, or mass spectrometry and other techniques. This systematic approach not only accelerates the discovery of high-yield strains but also provides key data feedback for the rational design of NRPS, effectively promoting the transformation process of LPs from basic research to industrial application.

### 5.3. Syn-BNP Strategies for Next-Generation Natural Product Discovery

**Figure 11:**
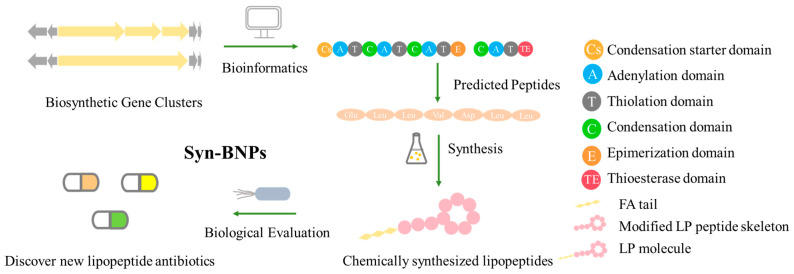
Schematic of the syn-BNP pipeline for discovering novel LPs targeting MDR bacteria from uncultivable microbial sources [76]. The workflow begins with the in silico prediction of novel biosynthetic pathways through bioinformatic analysis of extensive genomic datasets. The computationally identified NRPS gene clusters serve as blueprints for the subsequent chemical synthesis of the corresponding LPs. These synthetic compounds are then subjected to biological evaluation to identify novel antibacterial agents.

Leveraging bioinformatic algorithms and chemical synthesis to mine the structural information encoded in underutilized genetic resources, the synthetic bioinformatic natural product approach has emerged as a promising strategy in LP research. By analyzing the genomes of difficult-to-culture bacteria, researchers can predict the A domains within LP-like BGC and identify key substrate-binding residues, enabling the subsequent chemical synthesis of target LPs (Figure 11). This method has successfully yielded novel LPs such as SyCPAs, Macolacin, aquicidins, and Cilagicin, which exhibit potent activity against drug-resistant bacteria [76,77,78,119].

Compared to traditional chemical synthesis, its advantage lies in shifting the drug discovery paradigm from random screening to rational design, achieving leaps in efficiency, scope, and novelty. Furthermore, the design flexibility and ability to mine uncultivable resources intrinsic to syn-BNP facilitate rapid LP discovery and lead optimization. When combined with NRPS engineering to construct efficient microbial chassis, this integrated strategy paves the way for scalable production.

Despite its utility, the syn-BNP approach is limited by its dependence on sequence homology and simplistic modular logic, failing to account for NRPS 3D structure and dynamics. This impedes the accurate prediction of key features like FA incorporation and macrocyclization, resulting in synthetic compounds that diverge from natural products. Employing tools like AlphaFold2 for structural analysis and molecular simulations is crucial for refining these designs and bridging the functional gap [248].

### 5.4. AI-Driven Discovery of Novel LPs

The emerging topic of ML provides a powerful computational framework for novel LP discovery. By constructing predictive sequence-activity models, ML guides the rational design of LP libraries with enhanced biological properties, thereby reducing reliance on empirical screening and shortening development cycles. At the molecular discovery level, frameworks based on Graph Convolutional Networks, such as PmxPred, enable the efficient rational design of novel analogs and accurately predict their antimicrobial activity [249]. For production optimization, the integration of Artificial Neural Networks with Genetic Algorithms can significantly enhance the fermentation yield of specific LPs, such as Iturin A [250]. Furthermore, an intelligent “Design-Build-Test-Learn” cycle, driven by active learning and high-throughput metabolomics, allows for the global analysis and optimization of complex LP biosynthetic networks from a systems biology perspective [251]. These methods span the entire pipeline from the intelligent design of candidate molecules to intelligent industrial production, marking the entry of LP research and development into a new, data- and model-driven era.

**Figure 12:**
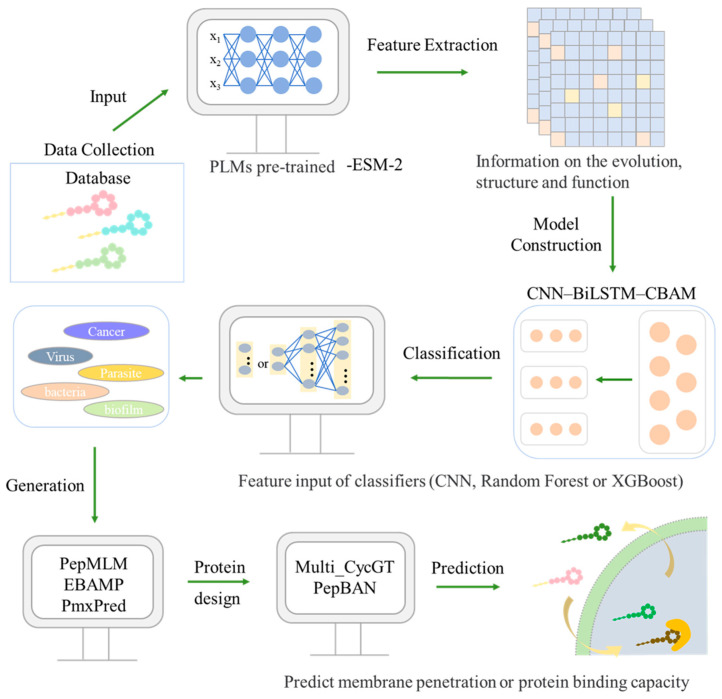
The schematic diagram of the process for discovering novel AMPs based on the Transformer. The process begins with the curation of a high-quality dataset of known AMP sequences. This dataset is used to train a Transformer model, which learns the underlying linguistic rules and structure–function relationships of AMPs. The trained model then generates a vast library of de novo peptide sequences predicted to possess antimicrobial properties. These candidate peptides were screened for toxicity and stability through silicon screening and finally synthesized for experimental verification of their antibacterial activity.

The application of AI in the rational design of LPs is currently in its nascent stages. Most existing AI tools and models have been developed for the broader category of AMPs, yet their frameworks hold significant translational potential for LP engineering (Figure 12). For instance, the random forest algorithm has been employed to screen key physicochemical features for the accurate prediction of anticancer peptide activity [252], enhance binding site prediction coverage and molecular docking success rates in interaction analysis [253,254], and construct models to effectively assess peptide binding affinity [255]. Neural network-based approaches have demonstrated powerful capabilities, enabling the mining of novel AMP sequences from the proteomes of extinct species [256] and achieving excellent performance in predicting cell-penetrating peptides using graph neural networks [257]. Tools like APPTEST facilitate high-precision structural resolution of short peptides [258,259], while LSTM-based generative models can construct targeted peptide libraries with high activity [260]. In bioactivity and safety assessment, multi-label deep learning models have significantly improved the prediction accuracy of peptide functions and toxicity [258,261,262]. Despite challenges such as high data demand, computational cost, and limited interpretability, neural networks remain a core driving force in peptide drug development. The prominent Transformer architecture, with its self-attention mechanism for capturing long-range dependencies, shows immense potential in property prediction, interaction analysis, and HTS of bioactive peptides. Key advances include: protein language models combined with classifiers for precise prediction of antimicrobial activity and cytotoxicity (the pLM4MRSA model) [263,264,265]; specialized architectures like Multi_CycGT for accurate prediction of cyclic peptide membrane permeability [266] and PepBAN for enhanced residue-level interaction analysis [267]; and generative models for large-scale de novo sequence design, such as PepMLM for generating high-specificity linear peptide binders [268] and the EBAMP framework for obtaining broad-spectrum AMPs with high experimentally validated activity [269]. Collectively, these advancements signify that AI has established a complete technological pipeline encompassing prediction, modeling, and design for peptide discovery, laying a robust computational foundation for the future rational design of LPs.

Looking ahead, the strategic integration of these complementary AI methodologies holds great promise for systematically accelerating the discovery and optimization of potent yet low-toxicity LPs.

Current rational design of LPs focuses predominantly on peptide sequence optimization, while largely overlooking the critical role of FA properties—including type, length, and modification—in determining bioactivity. A significant gap exists in systematic ML approaches for predicting and optimizing FA characteristics, with no publicly available tools reported.

A comparative analysis of the aforementioned methods for LP design and discovery reveals a clear paradigm shift in the field. Research is moving from single-technology-driven paradigms toward the deep integration of AI, bioinformatics, NRPS engineering, and chemical technologies. The development model is evolving from “random discovery” to SAR and ML, enabling the on-demand customization of LPs with target properties. Furthermore, the scope has expanded from “natural mining” to synthetic creation, leveraging synthetic bioinformatics and AI-generated designs to break natural structural constraints and create non-natural molecules with superior properties (Table 5). This series of transformations is poised to guide and accelerate the discovery of novel LP entities.

## 6. Applications of LPs Across Agriculture, Livestock, Healthcare, Petroleum, and Environmental Remediation

LPs, leveraging their unique combination of antimicrobial, surfactant, and biosignaling properties, are demonstrating transformative potential as green alternatives across multiple sectors. In crop cultivation, they transcend conventional pesticides by synergistically promoting plant defense and growth. This is evidenced by their potent antifungal activity-Chromorhipeptin derivative 1 with MIC of 0.04 μM against *Valsa mali*, ability to induce systemic resistance-iturin/fengycin against Fusarium head blight, and role in shaping beneficial rhizomicrobiomes—surfactin against banana wilt [270,271,272].

Field trials have demonstrated their effectiveness, with yield increases comparable to those achieved with chemical fungicides. For instance, B. velezensis CNPMS-22 increased maize yield to 8340.93 kg/ha [273]. In animal husbandry, they offer a solution to antibiotic resistance. *B. subtilis*-derived cyclic LPs linearly improve broiler growth and gut health, while surfactin modulates immunity, and probiotic strains like *B. velezensis* 24.5 show broad antimicrobial activity and gut adaptability [274,275,276]. In human medicine, specific LPs like DAP for MRSA/VRE infections and polymyxins for carbapenem-resistant G^−^ bacterial infections serve as critical last-resort antibiotics, necessitating strict use controls [277,278]. Within the petroleum industry, they function as efficient biosurfactants for enhanced oil recovery, with a surfactin mixture boosting heavy oil recovery by 31% [279]. For environmental remediation, they degrade persistent pollutants; *B. velezensis* MHNK1 with surfactin achieves 100% atrazine degradation in 4 days, and various LPs effectively remove 62–89.7% of oil hydrocarbons from contaminated matrices [280,281,282,283]. In summary, from safeguarding crops and livestock to treating critical infections, enhancing energy extraction, and restoring ecosystems, the multifaceted applications of LPs underscore their pivotal role in driving a sustainable and innovative bioeconomy.

## 7. Summary and Future Outlook

The development of LPs epitomizes a continuous evolutionary competition against bacterial resistance. Since their inception with polymyxins in the 1940s, LPs have been crucial in fighting drug-resistant infections due to their membrane-targeting action [284,285] and structural adaptability [233,286]. However, clinical use rapidly selects for resistance via membrane modification and efflux pumps, driving a paradigm shift from natural product screening to rational design [287].

Recent breakthroughs in elucidating SARs have enabled rational engineering of LP domains, yielding compounds with improved activity and therapeutic indices. While total chemical synthesis facilitates non-natural motif incorporation, its scalability is limited. Advances in combinatorial biosynthesis and NRPS engineering provide a robust platform for generating complex LP libraries in microbial hosts [220,288]. Innovations such as LP self-assembly into nanoparticles for targeted delivery and AI-integrated bioinformatic analysis pioneer data-driven discovery, guided by the principle of optimizing hydrophobicity-hydrophilicity balance to enhance membrane interaction and overcome resistance [210,289,290]. Beyond their core therapeutic role as a “last line of defense” against multidrug-resistant bacteria, LPs’ unique dual functionality has spawned a cross-sectoral ecosystem. They serve as biopesticides and biostimulants in agriculture, antibiotic alternatives in livestock feed, agents for enhanced oil recovery and pollution remediation in the petroleum industry, and degraders of persistent pesticides and hydrocarbons in environmental cleanup.

The future of LP development lies in converging four complementary strategies: precision chemical synthesis, NRPS engineering for natural scaffolds, AI-driven candidate screening, and universal nanocarrier delivery systems to enhance pharmacokinetics and safety. This integrated pipeline, from AI-led design to nano-enabled delivery, charts the course for next-generation LP-based therapeutics and broader industrial applications.

## Figures and Tables

**Table 2:** The antibacterial activity (MIC: μg/mL) and cytotoxicity (μg/mL) of DAP derivatives.

DAP Derivatives	Structure	MIC (μg/mL)			Cytotoxicity (μg/mL)	Reference
		*S. aureus*	*E. faecium*	*Streptococcus*		
DAP	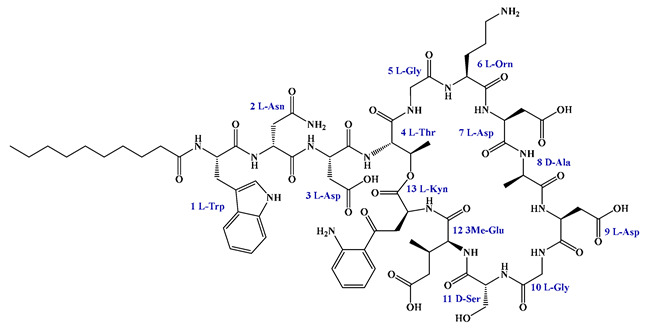	0.12–1	1–4	0.06–0.5	N.D.	[52]
Surotomycin	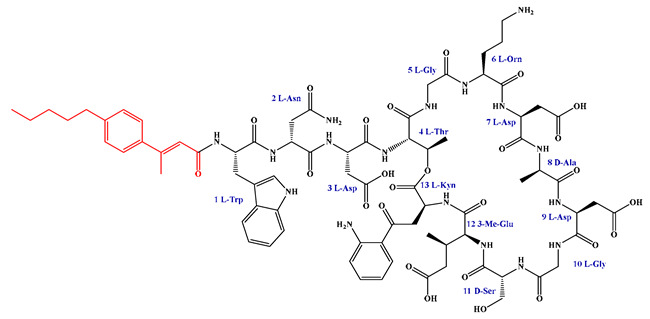	0.25–1	≤0.125–2	≤0.125–2	N.D.	[54]
Hexakynomycin	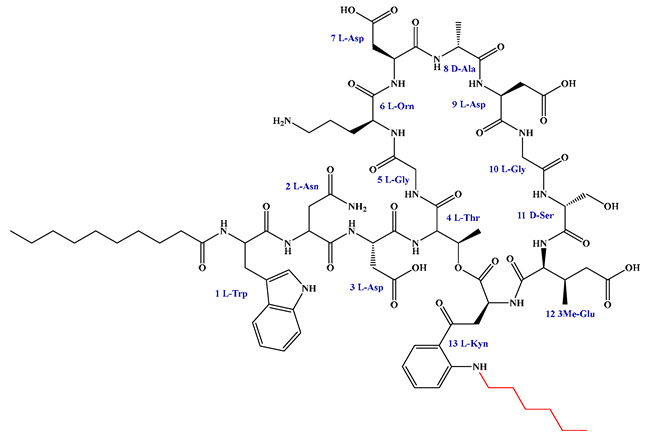	0.125–2 (2–8) *	0.25 (8) *	N.D.	N.D.	[55]
MX-2401	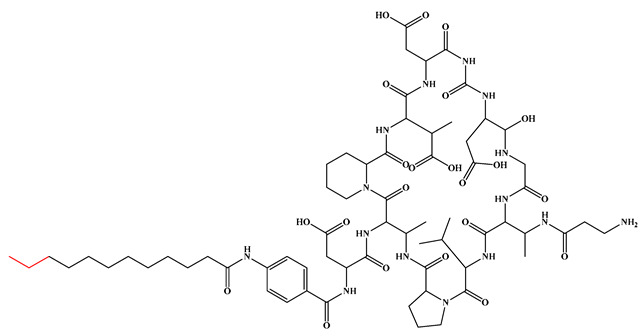	2 (1)	4 (4)	N.D.	N.D.	[56]
CB182122	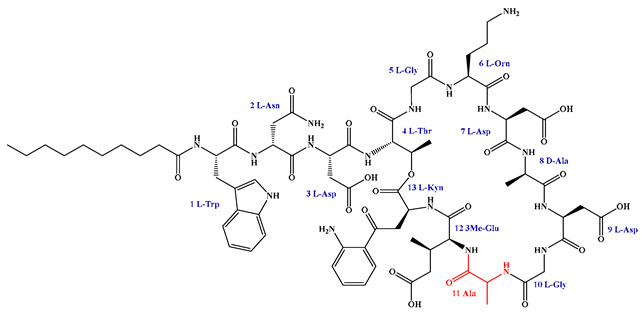	0.5–8 (1–16)	1–32 (2–64)	0.125 (0.125)	N.D.	[57]
DAP-8(Lys/Arg)-11(Arg)	/	0.13–1 (0.13–0.25) *	N.D.	N.D.	>128	[58]
Alkyl-Dap analogs(41)	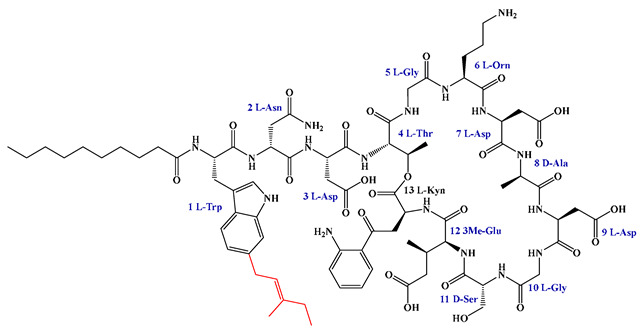	0.5–2.1 (0.6–8.1) *	0.1 (1.5) *	N.D.	Comparable to DAP	[59]
DAP-Me-Kyn-13(Kynomycin)	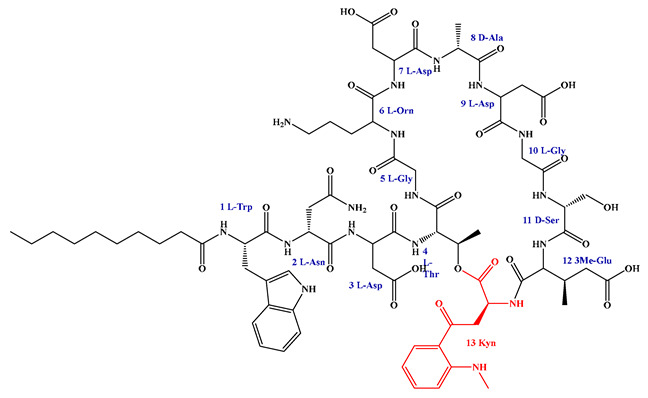	0.25–0.5 (0.5–2) *	1 (2–4)	0.5 (0.5)	>80 (>20)	[60]
DAP-1-Me-Trp-1	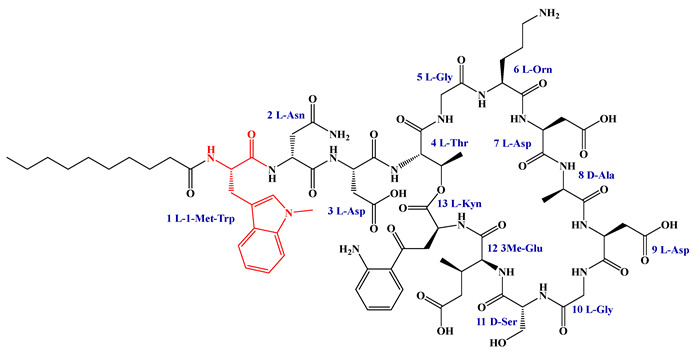	0.25–2 (0.5–2) *	1–2 (2–4)	N.D.	N.D.	[60]

* Represents clinical isolation of MDR (strains resistant to DAP and vancomycin/MRSA); () is the MIC of DAP; N.D. indicates no data.

**Table 5:** Comparison of LP structure design and modification methods.

Method	Basic Principle	Advantage	Drawback	Representative LPs
Structural rational design based on chemical synthesis	Based on structure–activity relationships, target LPs are directly constructed with precise control over each amino acid and lipid unit.	Extensive design flexibility Well-defined and high-purity products	High cost and synthetic complexity Poor scalability Heavy reliance on known SAR	BP475 and BP485 R-lycosin-I Biphenyl-Macolacin Biphenyl-Cilagicin
NRPS engineering	Genetic engineering is carried out on non-ribosomal peptide synthases responsible for LP biosynthesis, and the product structure is changed through methods such as module replacement and domain modification.	Enables production of complex LPs with defined stereochemistry. Potential for scalable production.	Technically complex Potentially very low yield Heavily reliant on native NRPS modules	Goadvionin A4 Iturin and rhizomide derivatization
HTS	Large LP libraries (chemically or biologically sourced) are constructed and rapidly screened via automated platforms to identify hit compounds.	May discover novel mechanisms of action Directly links to bioactivity data	Inefficient with low hit rates Dependent on library quality and diversity Hit-to-lead optimization is laborious	Brevistin S Surfactins Plipastatins
Syn-BNP	Predict LP structures from genomic data and chemically synthesize the resulting “virtual” molecules for activity validation.	Accesses nature’s “dark matter” Bypasses difficult heterologous expression Efficient	Prediction accuracy is not guaranteed Dependent on genomic data quality Challenging synthesis of complex structures remains	Macolacin Cilagicin
AI-powered discovery of novel LPs	Deep learning models learn the sequence–structure–activity relationships of known LPs to predict novel sequences with desired properties, which are then validated through synthesis.	Strong generative capability Rapid virtual screening of vast molecular libraries Multi-property optimization	Highly data-dependent Poor interpretability May generate infeasible or hard-to-synthesize molecules	Polymyxin derivatives

## Data Availability

No data was used for the research described in the article.

## Abbreviations

AMR	Antimicrobial resistance
AMPs	Antimicrobial peptides
AI	Artificial intelligence
A domain	Adenylation domain
BGCs	Biosynthetic gene clusters
Ca^2+^	Calcium ions
CL	Cardiolipin
CM	Cell membranes
Cs domain	Starter condensation domain
CW	Cell wall
C domain	Condensation domain
Dab	Diamino butyric acid
DAP	Daptomycin
E domain	Epimerization domain
FA	fatty acid
G^+^	Gram-positive
G^−^	Gram-negative
HTS	High-throughput screening
LNPs	Lipid nanoparticles
LP	Lipopeptide
LPS	Lipopolysaccharide
MALDI-TOF MS	Matrix-Assisted Laser Desorption/Ionization Time-of-Flight Mass Spectrometry
MDR	Multi-drug resistance
ML	Machine learning
MRSA	Methicillin-resistant *Staphylococcus aureus*
NRPS	Non-ribosomal peptide synthetase
OM	Outer membrane
PCP	Peptidyl carrier protein
PEA	Phosphatidylethanolamine
PMB	Polymyxin B
PKS	Polyketide synthases
PG	Phosphatidylglycerol
RiPP	Post-translationally modified peptide
ROS	Reactive oxygen species
RP-UPLC	Reversed-Phase Ultra-Performance Liquid Chromatography
RT-PCR	Real-Time Polymerase Chain Reaction
SAR	Structure–activity relationship
Syn-BNP	Synthetic-bioinformatic natural product
TE domain	Thioesterase domain
T domain	Thiolation domain
VRE	Vancomycin-Resistant *Enterococcus*
